# Exploring bovine brucellosis in Bangladesh: Current scenario and future outlook

**DOI:** 10.5455/javar.2024.k840

**Published:** 2024-12-27

**Authors:** Md. Zaminur Rahman, Palash Bose, Tanvir Ahamed, Papia Sultana, Md. Mukteruz-zaman, Kazi Abdus Sobur, Mst. Minara Khatun, Md. Ariful Islam

**Affiliations:** Department of Microbiology and Hygiene, Faculty of Veterinary Science, Bangladesh Agricultural University, Mymensingh, Bangladesh

**Keywords:** Control measures, economic impact, epidemiology, livestock health, one health approach, zoonotic disease

## Abstract

Bovine brucellosis is an enduring and formidable challenge in Bangladesh. In this first comprehensive review, we explored the historical, current, and future perspectives of bovine brucellosis outbreaks in Bangladesh. Data spanning from 1984 to 2023 regarding bovine brucellosis in Bangladesh were gathered from literature, reviews, conference papers, and online reports using various search engines and software tools. We considered 29 published documents and analyzed them thoroughly to evaluate the current status of bovine brucellosis for the present comprehensive review. We also suggest policy and other frameworks to mitigate and control the disease on a national scale. Bovine brucellosis in Bangladesh affects various livestock and poses economic burdens due to reduced milk and meat production with potential risks to human health. Over the past decade (2013-2023), the ruminant population in Bangladesh has increased substantially (between 1.46 and 26.95 million individuals), with goats showing the most significant growth, while financial losses due to bovine brucellosis have risen steadily, emphasizing its economic impact (basis on species between Euro currency 12.824 and 298.272). This review highlights brucellosis prevalence, diagnostic challenges, and traditional management practices contributing to its spread. Our findings indicate that bovine brucellosis was reported and prevalent in mild to severe forms across 26 districts of Bangladesh. Bangladesh has initiated measures such as vaccination and awareness campaigns, but effective control remains challenging due to diverse farming systems and resource constraints. A one-health approach is advocated for future strategies, emphasizing community engagement and multidisciplinary efforts to address the complex challenges posed by bovine brucellosis in Bangladesh, ultimately aiming to safeguard livestock health, public health, and economic stability.

## Introduction

Bovine brucellosis, a persistent bacterial infection caused by *Brucella* species, poses significant threats to livestock health and zoonotic transmission risks to humans, thereby impacting global agricultural practices [1]. Within Bangladesh, a nation defined by its densely populated terrain, burgeoning livestock sector, and intricate web of socioeconomic factors, bovine brucellosis outbreaks have emerged as a pressing concern, presenting multifaceted challenges and profound implications [2]. The story of bovine brucellosis in Bangladesh dates back to when the nation was known as East Pakistan, preceding its independence in 1971. During this period, bovine brucellosis existed, yet it remained largely underreported. In the 1970s and 1980s, sporadic outbreaks of the disease were documented, but knowledge and resources for a robust response were limited [3].

A pivotal moment arrived in the early 1990’s when Bangladesh initiated comprehensive studies to assess the prevalence of bovine brucellosis [4]. These investigations revealed a troubling reality: the disease not only persisted but spread across various parts of the nation, posing serious threats to both livestock production and human health [5,6]. This moment marked the beginning of dedicated efforts to better understand the disease, leading to the creation of strategies for its control and mitigation [7]. The disease persists, impacting not only cattle but also buffalo and small ruminants, resulting in diminished milk and meat production, reproductive issues, and costs associated with disease diagnosis and control measures [2,8]. Moreover, bovine brucellosis poses a formidable threat to public health, with the potential for transmission to humans through the consumption of contaminated dairy and meat products [9].

Bovine brucellosis in Bangladesh presents a challenge characterized by several significant features. First, its prevalence is widespread, with varying rates across different regions, making it a nationwide concern that transcends geographical boundaries [10]. Accurate diagnosis remains a daunting task due to the limited availability of well-equipped diagnostic facilities and trained personnel in many areas, hindering effective monitoring and control efforts. Traditional livestock management practices, such as communal grazing and unregulated animal movement, further contribute to the persistence and spread of the disease [11]. Notably, bovine brucellosis has zoonotic implications, posing a risk to individuals involved in the livestock industry, particularly farmers and dairy workers [12]. The decrease in milk and meat production, coupled with rising healthcare costs, exacerbates the issue, emphasizing the urgent need for comprehensive strategies to combat this pervasive and debilitating disease [13].

In response to these challenges, Bangladesh has initiated a series of measures to combat bovine brucellosis, including vaccination campaigns, increases in diagnostic capacity, and public awareness programs [2]. However, the path toward effective disease control remains unclear, given the diversity of livestock farming systems, resource constraints, and the imperative for sustainable strategies [14]. Against this backdrop, we discuss the future of bovine brucellosis in Bangladesh, seeking to glean insights that can guide the nation toward a more secure and prosperous future [5,15].

In our study of bovine brucellosis outbreaks in Bangladesh, we focus on several important questions. First, we look at how the disease has changed over time and why it wasn’t reported accurately in the past. Second, we examine how common the disease is now and how it spreads, and we also see how it affects milk and meat production and the country’s need for expensive imports. Third, we study how the disease can affect people, especially those who work with livestock, and how it can impact public health and food safety. Fourth, we look at the challenges of diagnosing the disease correctly and what improvements can be made to testing methods. Fifth, we explore how traditional ways of raising animals might make the disease spread more easily and think about ways to balance tradition with controlling the disease. Sixth, we consider how much money is lost because of the disease, including how it affects farmers’ incomes and the country’s economy. Finally, we stress the importance of working together in different fields to find long-term solutions that work for everyone, considering the different ways livestock are raised and the resources available.

In all aspects, we evaluate the historical context of the disease, particularly focusing on its evolution post-independence and the factors influencing past underestimations of prevalence. Additionally, we quantify contemporary prevalence and distribution across various livestock species, alongside its associated economic burden on production. Finally, we explore current transmission dynamics within and between herds, emphasizing the potential role of traditional livestock practices in perpetuating the issue. Through this investigation, we strive to gain comprehensive insights into the multifaceted nature of bovine brucellosis in Bangladesh.

## Methodology

**Figure 1.**
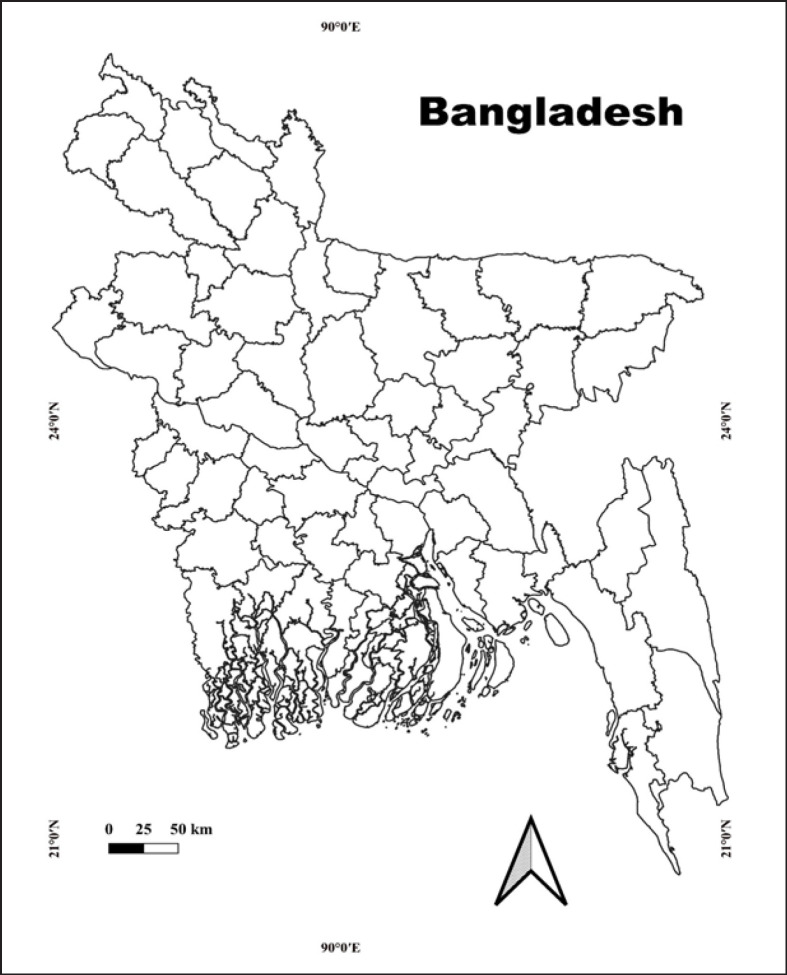
The review covered different districts of Bangladesh.

The record of bovine brucellosis in different animals from different districts of Bangladesh was taken into consideration for this study (Fig. 1). The selection criteria for this review included published articles or theses reporting data or results on bovine brucellosis within Bangladeshi territory, specifically mentioning bovine species, and addressing any aspect of brucellosis. These sources had to be available on the internet, ensuring accessibility and verification. Only peer-reviewed and finalized research was considered, thus excluding conference abstracts, data presented at conferences, and preprints. The list of published papers, including their publication years, study areas, key findings, and types of animals studied, is provided in Supplementary Table 1.

**Figure 2.**
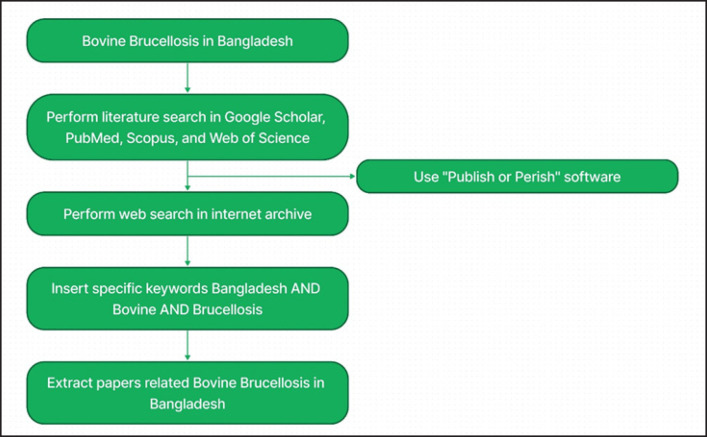
Literature search strategy for the current study.

These data were accumulated from the existing literature, monographs, reviews, checklists, catalogs, posters, conference papers, conference posters, websites, and livestock reports from Bangladesh from the periods 1984 to 2023. The literature search was performed with Google Scholar, PubMed, Scopus, Web of Science, and web search using “Publish or Perish” software Harzing [16] from the period of May 2023 and October 2023 (Fig. 2). Another web search in the internet archive was also carried out. The specific keywords were inserted and searched for the documents Bangladesh AND Bovine AND Brucellosis. We extracted the papers related to bovine brucellosis in Bangladesh.

## Historical Overview of Bovine Brucellosis in Bangladesh

### Pre-independence era

**Figure 3.**
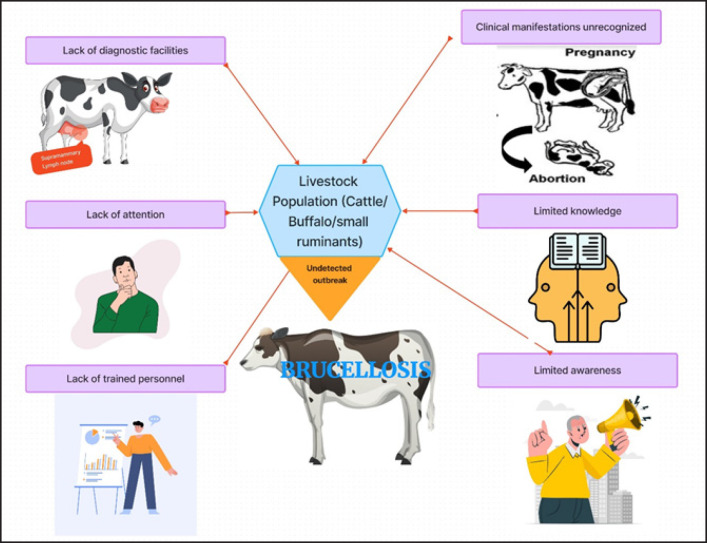
Reasons for the hidden bovine brucellosis in the pre-independence periods.

Bovine brucellosis, caused by the bacterium *Brucella abortus*, existed in the region that is now Bangladesh well before the nation’s independence in 1971. However, during this pre-independence period, the disease remained largely inconspicuous and underreported. It lurked within the livestock population, affecting cattle, buffalo, and small ruminants, but without attracting significant attention [17]. Several factors contributed to the disease’s underreported prevalence during this time. Foremost was the limited awareness and knowledge of bovine brucellosis among both the farming communities and the authorities [5]. The disease’s clinical manifestations in animals, including reproductive issues such as abortions, stillbirths, and reduced fertility, often went unrecognized or were attributed to other causes (Fig. 3). Moreover, the lack of well-established diagnostic facilities and trained personnel meant that bovine brucellosis cases remained largely undetected. In many rural areas, where livestock farming was prevalent, diagnostic resources were scarce, and farmers had little access to tools that could identify the disease. This diagnostic deficit perpetuated a veil of obscurity around the disease, allowing it to circulate silently within the livestock population [2,18]. As a consequence, bovine brucellosis during this period remained a “hidden” threat, largely unacknowledged in official records and public consciousness.

### Post-independence era

The early 1970s and 1980s witnessed some documented cases that served as a harbinger of the disease’s tenacious presence [3]. The transition to an independent nation meant an intensified focus on agriculture and livestock development [19]. As Bangladesh sought to secure its food production and economic stability, the livestock sector expanded, increasing the population of cattle, buffalo, and small ruminants [20]. With this growth came an enhanced scrutiny of livestock health, which, in turn, led to the identification of bovine brucellosis cases that had previously escaped notice [21].

**Figure 4.**
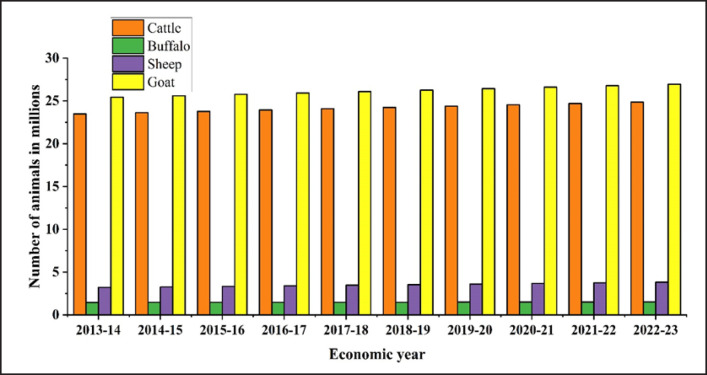
The farmed and household ruminant animal statistics from 2013-2023.

The household and farmed animal statistics of the Ministry of Fisheries and Livestock suggested that, over the 10 years (2013-2023), there has been a consistent increase in the populations of cattle (23.488 million–24.856 million), buffalo (1.457 million–1.516 million), sheep (3.206 million–3.827 million), and goats (25.439 million–26.945 million), with goats experiencing the most significant growth among the four species [22] (Fig. 4).

The post-independence era in Bangladesh brought with it a newfound awareness of bovine brucellosis as sporadic outbreaks began to surface. Furthermore, there was a growing realization of the economic implications of bovine brucellosis [23]. Livestock, particularly cattle, play a pivotal role in the livelihoods of rural communities, contributing significantly to milk and meat production [20]. The economic losses incurred due to reduced productivity, reproductive issues in animals, and the associated healthcare costs began to draw attention [24]. The combination of these factors spurred efforts to gain a deeper understanding of the disease. Bangladesh initiated a series of comprehensive studies in the early 2000s to assess the prevalence and distribution of bovine brucellosis [25]. These investigations marked a turning point in the nation’s approach to the disease. They revealed a disconcerting reality: bovine brucellosis had not merely persisted but had proliferated across diverse corners of the nation [10].

Meanwhile, the risk of zoonotic transmission loomed large, with those engaged in the livestock sector, particularly farmers and dairy workers, at risk of contracting the disease through close contact with infected animals or the consumption of contaminated dairy and meat products [26]. This juncture marked the genesis of dedicated efforts to combat bovine brucellosis in Bangladesh. The nation had transitioned from a state of relative unawareness to one where the disease was recognized as a pressing concern with far-reaching implications [13]. As Bangladesh embarked on a journey to confront this formidable challenge, the historical context of bovine brucellosis served as a crucial backdrop, informing current and future perspectives on disease management and control [27].

## EPidemiology of Bovine Brucellosis in Bangladesh

### Prevalence and distribution

The prevalence of bovine brucellosis in Bangladesh is a subject of concern and study. The disease affects not only cattle but also buffalo and small ruminants, amplifying its impact on livestock productivity and public health [8]. The prevalence rates vary across regions, making bovine brucellosis a nationwide concern due to the diverse livestock farming systems in Bangladesh, from large commercial farms to smallholder and subsistence farming [10,28]. In commercial farms, management practices are often more controlled, with better biosecurity measures in place [29]. This can limit disease transmission, leading to lower prevalence rates. In contrast, smallholder farms may practice communal grazing and have limited resources for disease control, creating an environment conducive to disease spread [30].

Furthermore, the geographical distribution of bovine brucellosis is influenced by the movement of livestock [31]. Unchecked animal movements, common in traditional livestock husbandry practices, contribute to the dissemination of the disease within and between herds [32]. Studies have revealed varying prevalence rates, with some regions experiencing higher rates of infection than others. The northern and western regions, characterized by intensive livestock farming, have reported higher prevalence rates [33].

### Diagnostic challenges

**Figure 5.**
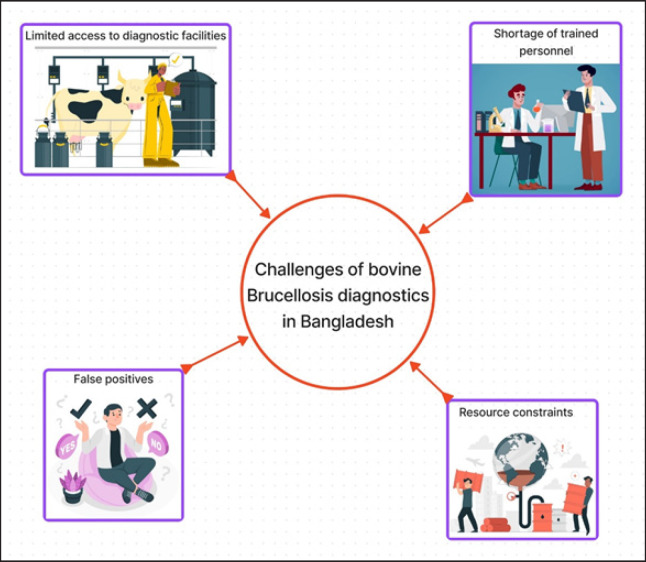
Some major challenges in diagnostics of bovine brucellosis in Bangladesh.

Accurate diagnosis is a cornerstone of bovine brucellosis management, but it remains a formidable challenge in Bangladesh [8]. The diagnostic methods for bovine brucellosis typically involve serological tests, such as the rose bengal plate test (RBPT) and the serum agglutination test (SAT). These tests detect antibodies produced by infected animals [34]. However, cross-reactivity with other pathogens and vaccines can lead to false-positive results, complicating the interpretation of test outcomes [35]. Moreover, the availability of diagnostic infrastructure is unevenly distributed across the country. In urban and peri-urban areas, diagnostic facilities are relatively accessible. Still, in remote rural regions, where a significant proportion of livestock farming takes place, there is a glaring lack of resources. The scarcity of well-equipped diagnostic facilities and trained personnel, particularly in rural areas where livestock rearing prevails, hampers effective disease monitoring and control [35]. Farmers in these areas often lack access to timely and accurate diagnostic services. The inadequacy of diagnostic infrastructure contributes to the disease’s underreporting, as unidentified cases serve as reservoirs of infection. The lack of a robust surveillance system further exacerbates this challenge, as the true extent of the disease remains elusive. Many rural areas lack well-equipped diagnostic laboratories, restricting access to serological and molecular tests. This limitation hampers disease surveillance and control efforts, particularly in remote regions. The scarcity of skilled laboratory technicians and veterinarians proficient in Brucellosis diagnosis is a significant constraint. This shortage impacts the quality and coverage of diagnostic services. Serological tests, particularly RBPT, can yield false-positive results due to cross-reactivity with other pathogens. Confirmatory tests such as serum tube agglutination test (STAT) and complement fixation test (CFT) are essential to minimize false positives [36]. Procuring diagnostic kits and maintaining the necessary laboratory infrastructure demands financial resources that may not always be readily available, hindering consistent testing (Fig. 5).

### Risk factors

**Figure 6.**
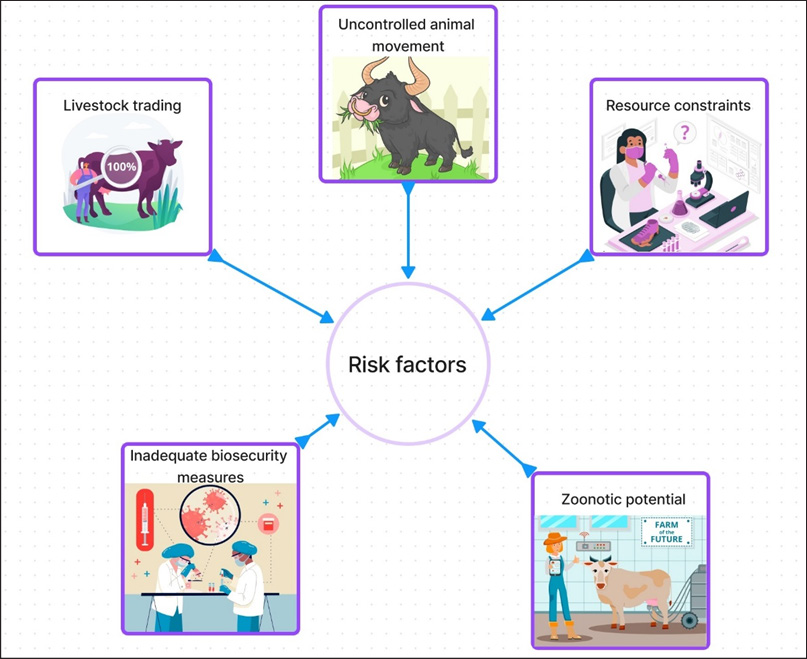
Some risk factors for the prevalence of bovine brucellosis in Bangladesh.

Several risk factors contribute to the epidemiology of bovine brucellosis in Bangladesh. Understanding these factors is essential for designing targeted control measures. Key risk factors are presented in Figure 6.

Traditional livestock management practices, such as communal grazing and unregulated animal movement, facilitate the transmission of bovine brucellosis within and between herds, while limited resources for disease control on smallholder farms often result in inadequate biosecurity measures, including the absence of quarantine facilities and the mingling of animals from different sources [37,38]. The buying and selling of animals in markets and across regions contributes to disease dissemination. Infected animals introduced into new herds can spark outbreaks [39]. The zoonotic potential of bovine brucellosis poses a risk to public health. Individuals engaged in the livestock sector, including farmers and dairy workers, are at risk of contracting the disease through close contact with infected animals or the consumption of contaminated dairy and meat products [31]. Resource limitations, including financial constraints, impact the ability to implement disease control measures effectively. Vaccination campaigns, diagnostic testing, and public awareness programs all require financial resources [27].

### Challenges and future perspectives

**Figure 7.**
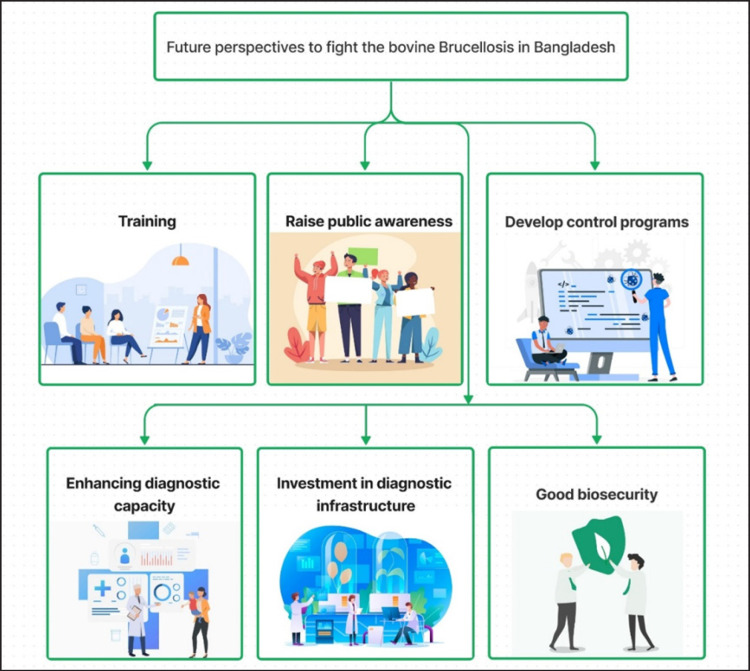
Major challenges and future perspectives to fight the bovine brucellosis in Bangladesh.

The epidemiology of bovine brucellosis in Bangladesh is characterized by complexity, shaped by diverse livestock farming systems, resource constraints, and traditional practices. Achieving effective disease control necessitates a multifaceted approach that addresses these challenges. Future perspectives on bovine brucellosis in Bangladesh must include strategies for enhancing diagnostic capacity, particularly in rural areas where the disease is prevalent. Investment in diagnostic infrastructure and the training of personnel is imperative for accurate disease surveillance and monitoring. Additionally, a concerted effort to raise public awareness about the disease’s zoonotic potential and the importance of safe food practices is essential. Engaging with farming communities and promoting effective biosecurity measures can help reduce disease transmission. Moreover, the development of cost-effective and region-specific control programs, including vaccination campaigns, will be critical (Fig. 7).

## Control and Prevention Measures

### Vaccination programs

Vaccination can play a pivotal role in controlling bovine brucellosis. In Bangladesh, efforts have been made to implement vaccination programs aimed at reducing the prevalence of the disease among cattle, buffalo, and small ruminants [27]. One of the vaccines used in bovine brucellosis control is the live attenuated strain, *Brucella abortus* strain 19 (S19). Live attenuated, inactivated, genetically modified deoxyribonucleic acid (DNA) subunit vaccines and vector vaccines are used in several developed countries. Vaccination with *B. abortus* strain 19 vaccine has been practiced in some developed countries [40]. However, the introduction of S19 and RB51 vaccines has the potential to improve the overall effectiveness of vaccination programs in Bangladesh, but there is no legislation to administer this vaccine [41]. The RB51 vaccine is a naturally occurring rough mutant, deficient in lipopolysaccharide O-antigen, and derived from the virulent smooth strain *B. abortus* 2308 [42]. Moreover, the challenges of these vaccines under field conditions in aspects of Bangladesh are still numerous. However,* B. abourtus* *biovar* 3 has already been isolated from dairy cattle in Bangladesh, and an inactivated vaccine, if developed, could be effective. The isolation of *Brucella abortus* biovar 3 in Bangladesh is crucial for effective disease management and control, as it aids in tracking the spread and prevalence of bovine brucellosis. This zoonotic pathogen poses a significant public health risk, with potential economic impacts due to decreased livestock productivity. Identifying this specific biovar informs vaccination strategies, ensuring that they are effective against local strains. Additionally, it provides valuable data for scientific research, contributing to better diagnostic and therapeutic approaches to combat brucellosis in both animals and humans [21].

However, this vaccine has its limitations, including the potential to cause abortions in pregnant animals and the interference with diagnostic tests, leading to false-positive results [35]. To address these limitations, efforts have been made to introduce a safer and more effective vaccine known as RB51. RB51 is a rough mutant strain of *B. abortus* that does not cause abortions or interfere with diagnostic tests [42]. The introduction of RB51 has the potential to improve the overall effectiveness of vaccination programs in Bangladesh. Despite these vaccination efforts, challenges remain. Ensuring that vaccines reach all regions of the country, including remote rural areas where many smallholder farmers reside, is a logistical challenge. Furthermore, the sustainability of vaccination programs hinges on adequate funding, cold chain maintenance, and community participation [43].

### Diagnostic improvements

Accurate and timely diagnosis of bovine brucellosis is essential for effective disease control in Bangladesh, where ongoing efforts to improve diagnostic methods are crucial for enhancing disease surveillance and monitoring [44]. A key development is the establishment of regional diagnostic laboratories, fully equipped with necessary facilities and staffed by trained personnel [35]. These laboratories are pivotal in diagnosing the disease and conducting confirmatory tests, lessening dependence on central facilities and enhancing accessibility for farmers across different regions [27]. Furthermore, research has been conducted on advanced diagnostic methods, including polymerase chain reaction (PCR) assays [15]. PCR-based tests offer higher specificity and sensitivity than traditional serological tests, potentially decreasing false-positive results and enhancing disease detection capabilities [45]. Ongoing research aims to develop more specific and reliable serological tests to overcome cross-reactivity with other pathogens and vaccines, crucial for distinguishing between vaccinated and infected animals in effective disease management [46]. Furthermore, mobile diagnostic units can be deployed in some areas to provide on-site testing and diagnostic services to farmers.

### Public awareness campaigns

Public awareness campaigns have been launched to educate livestock farmers and the general public about the risks associated with bovine brucellosis and the importance of disease prevention measures [47]. These campaigns aim to raise awareness about the zoonotic potential of bovine brucellosis, highlighting the risks faced by individuals engaged in the livestock sector, such as farmers and dairy workers [27]. Information is disseminated through various channels, including radio broadcasts, community meetings, and printed materials. Farmers are educated about the signs of bovine brucellosis in animals, emphasizing the importance of early detection and reporting [18]. They are also encouraged to adopt good biosecurity measures, such as segregating infected animals and practicing proper hygiene when handling animals or animal products [48]. In addition to raising awareness among farmers, public awareness campaigns also focus on the importance of safe food practices. Consumers are informed about the risks of consuming contaminated dairy and meat products and are encouraged to purchase products from reputable sources.

### Sustainable control measures

Sustainable control measures are imperative to address the long-term challenges posed by bovine brucellosis in Bangladesh. These measures consider the diversity of livestock farming systems, resource constraints, and the need for effective disease control [9]. One approach to sustainability is the development of region-specific control programs. Tailoring control strategies to specific contexts can improve their effectiveness [49]. The engagement of communities and farmers in disease control efforts is another key aspect of sustainability [13]. Farmers are encouraged to actively participate in disease monitoring, reporting, and control measures. Community-based surveillance systems have been proposed to involve local communities in disease detection and reporting, enabling a more rapid response to outbreaks [50]. Efforts to strengthen the cold chain infrastructure for vaccine distribution are essential to ensure the availability and efficacy of vaccines in remote areas, as ensuring that vaccines are stored and transported at the correct temperatures is critical to their success in disease control [51]. Additionally, collaboration between government agencies, non-governmental organizations (NGOs), research institutions, and international partners is essential for the sustainable control of bovine brucellosis.

### Challenges and future directions

While progress has been made in controlling bovine brucellosis in Bangladesh, challenges persist. Adequate funding and resource allocation are essential to sustain and expand control efforts. The logistical challenges of vaccine distribution, especially in remote areas, need to be continually addressed [27]. Future directions should prioritize research into improved diagnostic methods, the development of more effective vaccines, and region-specific control strategies.

## Public Health Implications

### Occupational hazards for livestock workers

Farmers and livestock workers in Bangladesh, whose livelihoods depend on close contact with animals, are at increased risk of bovine brucellosis infection. Occupational exposure to infected animals, contaminated animal products, and the birthing materials of infected animals places them at considerable risk [9]. This occupational hazard has far-reaching consequences for both the individuals involved and the broader community [1]. The pathogen can enter their bodies through cuts, abrasions, or mucous membranes, leading to chronic or acute infection [15]. This not only endangers their health but also disrupts their ability to work and support their families. Furthermore, the economic consequences of bovine brucellosis extend to livestock workers. Infected animals experience reduced fertility and milk production, impacting the income and livelihoods of those dependent on these animals [52].

### The zoonotic threat

Bovine brucellosis in Bangladesh has significant zoonotic potential, posing a direct threat to public health [2]. Zoonoses are diseases that can be transmitted from animals to humans, and bovine brucellosis is one such zoonotic disease [13]. The primary route of zoonotic transmission is through the consumption of contaminated dairy and meat products [47]. In Bangladesh, where dairy and meat play a central role in the daily diet, the risk of exposure to *Brucella* is substantial [53]. Raw milk, in particular, carries a high risk when obtained from infected animals. Traditional practices such as consuming raw milk, curd, or milk-based sweets are common, making it easier for the pathogen to infiltrate human populations [47]. Human infection with *Brucella* can lead to a debilitating disease known as human brucellosis. The symptoms of human brucellosis include fever, fatigue, joint pain, and muscle pain, which can persist for prolonged periods. These symptoms not only compromise an individual’s quality of life but also hinder their ability to work and contribute to society [54]. Beyond the physical toll, human brucellosis can also impose a significant economic burden on individuals and healthcare systems. Patients often require prolonged antibiotic treatments, and complications such as endocarditis, arthritis, or neurological issues can arise, necessitating specialized medical care [55]. The economic impact extends to healthcare expenditures, making it essential to consider the broader financial ramifications of the disease.

### Implications for food safety

The nexus between bovine brucellosis and food safety in Bangladesh is a critical concern [47]. Contaminated dairy and meat products pose a direct risk to consumers, potentially leading to widespread outbreaks of human brucellosis [47]. Traditional methods of milk and meat preparation, which often involve minimal heat treatment or raw consumption, exacerbate the food safety risk [56]. Consumers are often unaware of the potential contamination of dairy and meat products with *Brucella*, as the pathogen does not alter the taste, color, or odor of these products [57]. This lack of visual or sensory cues makes it challenging for consumers to detect the presence of the pathogen, rendering them unwitting carriers of the disease [58]. Ensuring food safety in the context of bovine brucellosis necessitates a multifaceted approach. It requires stringent hygiene measures during the collection, processing, and sale of dairy and meat products. Furthermore, promoting public awareness of the risks associated with consuming raw or inadequately processed products is crucial. Key measures involve ensuring all dairy products undergo proper pasteurization, educating consumers and food handlers on thoroughly cooking meat to internal temperatures above 63°C (145°F), and implementing strict sanitation protocols in slaughterhouses and dairy farms. Establishing robust traceability systems to track product sources, conducting regular testing of livestock, launching public awareness campaigns about the risks of raw or inadequately processed products, providing comprehensive training for food handlers, and strengthening regulatory oversight and enforcement of food safety standards are also essential to reduce *Brucella* contamination and protect public health.

### The role of government and public health authorities

To address the public health implications of bovine brucellosis, a collaborative effort between government agencies, public health authorities, and the livestock sector is imperative. Government agencies must lead in developing and implementing comprehensive policies and regulations aimed at mitigating the zoonotic threat. One key strategy is the enforcement of food safety regulations that mandate proper milk pasteurization and meat cooking standards [59]. Rigorous inspection and testing of dairy and meat processing units can help ensure compliance. Public health authorities can play a vital role in monitoring food safety standards and responding to outbreaks of human brucellosis [53]. In addition to regulatory measures, public health authorities should engage in public awareness campaigns. These campaigns should educate consumers about the risks associated with consuming raw dairy and meat products, as well as promote safe food handling and hygiene practices in households [60].

**Figure 8.**
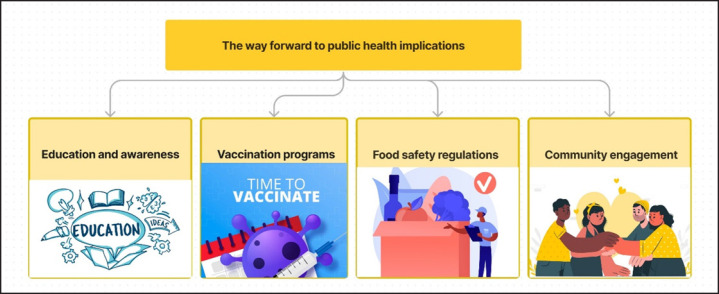
The way forward to public health implications for the bovine brucellosis in Bangladesh.

Addressing the public health implications of bovine brucellosis in Bangladesh requires a multifaceted approach that encompasses not only the livestock sector but also public health, food safety, and community engagement. Enhanced surveillance systems can help detect outbreaks of human brucellosis and trace them back to their animal sources. Timely detection is critical for initiating control measures (Fig. 8). Public awareness campaigns should be intensified to inform consumers, farmers, and livestock workers about the risks associated with bovine brucellosis and the importance of food safety measures. Stringent food safety regulations should be enforced, mandating proper pasteurization and cooking standards for dairy and meat products. Expanding vaccination programs for livestock can reduce the prevalence of bovine brucellosis, thereby reducing the zoonotic risk. Engaging local communities in disease monitoring and control efforts can empower them to take proactive measures to prevent bovine brucellosis.

## Public Health Implications

### Serological tests

Serological tests are the cornerstone of bovine brucellosis diagnosis in Bangladesh, as they enable the detection of antibodies produced by the host in response to *Brucella* infection [2]. Several serological tests have been employed in the country’s diagnostic arsenal. RBPT is one of the most commonly used tests for the preliminary screening of bovine brucellosis in Bangladesh [2]. It is an agglutination test that detects antibodies against *Brucella* in serum samples. While RBPT is cost-effective and relatively simple to perform, it has limitations, including low specificity, which can result in false-positive results [45]. The STAT is another widely used serological test in Bangladesh. It is more specific than RBPT and is often employed as a confirmatory test following a positive RBPT result. STAT quantifies antibody titers and is considered more reliable, but it requires a higher level of laboratory expertise [61]. CFT is a highly specific test used for confirmatory diagnosis. It measures the ability of antibodies to fix complement, providing quantitative results. CFT is valuable in distinguishing between true infections and false positives but demands a well-equipped laboratory and trained personnel [62]. Indirect enzyme-linked immunosorbent assay (ELISA) and competitive ELISA have gained popularity in recent years for their sensitivity and specificity. These tests detect specific antibodies to *Brucella* and are suitable for large-scale screening. They offer advantages in terms of automation and higher throughput but require laboratory infrastructure and skilled technicians [63].

In Bangladesh, the surveillance of brucellosis in goats reveals a relatively low prevalence in selected areas, highlighting the need for enhanced monitoring strategies to track and manage the disease effectively [64]. Meanwhile, research using the indirect enzyme-linked immunosorbent assay (iELISA) technique identified a modest 1.5% seroprevalence of *Brucella* in dairy cattle, underscoring the importance of robust diagnostic methods to accurately assess disease burden and inform targeted intervention efforts. Moreover, the higher seroprevalence of human brucellosis among high-risk groups underscores the urgent need for preventive measures to protect vulnerable populations from the potential health impacts of zoonotic transmission [5].

The significant economic impact of brucellosis in Bangladesh’s Mymensingh district underscores the imperative for proactive control strategies to mitigate financial losses and safeguard livelihoods [65]. Concurrently, the study revealing a 6.6% seroprevalence of brucellosis in cattle further highlights the widespread presence of the disease within the country’s livestock populations, necessitating targeted control measures to curb its spread [44]. Additionally, the detection of *Brucella abortus* in both humans and domestic ruminants underscores the interconnectedness of disease transmission pathways, emphasizing the need for integrated surveillance systems that encompass multiple species [33].

Milk ring test (MRT) is employed for the detection of brucellosis in milk samples. It is especially useful for dairy herds, where testing milk is more convenient than blood. MRT, however, has limitations in sensitivity, and positive results should be confirmed with other serological tests [66]. The methods for diagnosis of bovine brucellosis are presented in Table 1.

**Table 1.** List of some available tests for the diagnosis of brucellosis [67].

Tests	Agglutination tests	Primary binding assays
Slow	Slow Agglutination (SAT)	Radioimmunoassay
	SAT with added reducing agents such as 2- mercaptoethanol or dithiothreitol	Fluorescence immunoassay
	SAT with the addition of rivanol to precipitate glycoproteins	Particle counting fluorescence immunoassay
	SAT with the addition of ethylene diamine tetraacetic acid to reduce IgM binding (EDTA)	Indirect enzyme immunoassay
	SAT with antiglobulin added to enhance agglutination	Competitive enzyme immunoassay
	Milk ring test	Fluorescence polarization assay
Rapid	Rose bengal	
	Modified rose bengal	
	Buffered plate agglutination	
	Card	
	Heat treatment of serum	
	Addition of 10% sodium chloride	
Tests	Precipitation tests	Compliment fixation test
	Agar gel immunodiffusion	Warm
	Radial immunodiffusion	Cold
		Hemolysis in gel
		Indirect hemolysis
Tests	Allergic tests	
	Skin test	

### Molecular diagnostics

While serological tests are vital for diagnosing bovine brucellosis, molecular diagnostic techniques offer additional specificity and can detect the presence of *Brucella* DNA [68]. PCR is a molecular method that has shown promise in Bangladesh’s context. PCR assays target specific regions of *Brucella* DNA, allowing for the detection of the bacterium even in the absence of detectable antibodies. Real-time PCR is particularly valuable for its speed and sensitivity [69]. However, molecular diagnostics like PCR require well-equipped laboratories, specialized equipment, and skilled personnel, making them less accessible in rural areas.

### Advancements and initiatives

**Figure 9.**
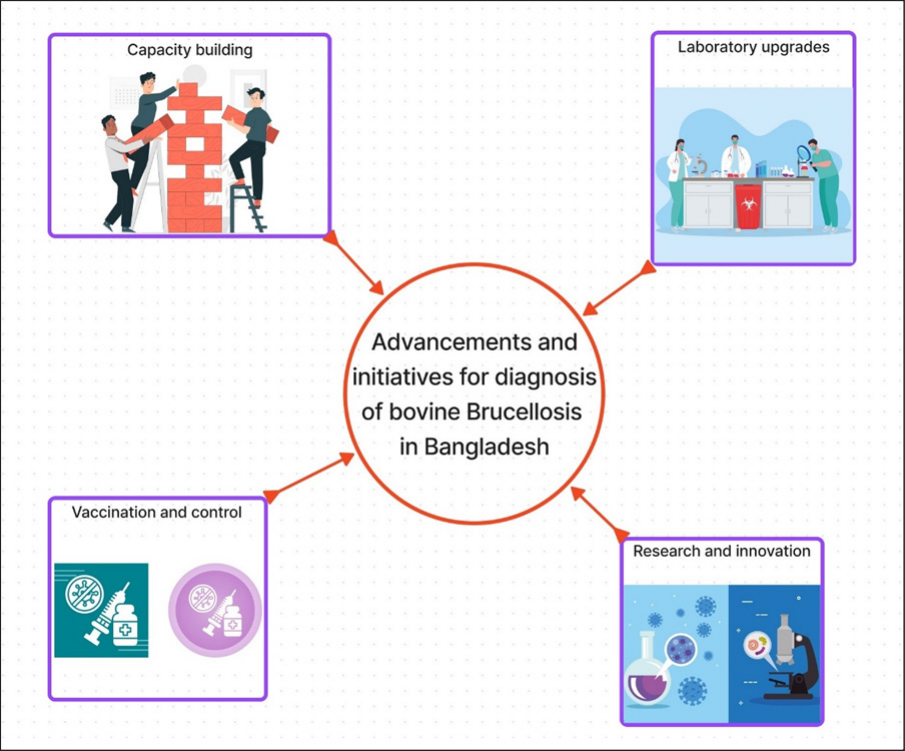
Advancements and initiatives for diagnosis of bovine brucellosis in Bangladesh.

Training programs for laboratory technicians and veterinarians have been conducted to enhance diagnostic proficiency. These initiatives aim to expand the pool of skilled personnel capable of conducting accurate tests [13]. Investment in laboratory infrastructure and equipment has been prioritized to improve diagnostic capabilities. This includes establishing brucellosis diagnostic laboratories in different regions, especially in areas with high livestock populations [27]. The implementation of vaccination programs, particularly for replacement heifers, aims to reduce the prevalence of bovine brucellosis. Such programs are essential for lowering the disease burden and minimizing the number of positive animals that need diagnostic testing. Research initiatives have explored the development of cost-effective, field-applicable diagnostic tools suitable for resource-constrained settings. These innovations hold promise for improving diagnostics in rural areas. Efforts should focus on extending diagnostic services to underserved rural areas, including the establishment of mobile diagnostic units. Telemedicine and remote diagnostic support can also be explored. Raising awareness among livestock farmers about the importance of testing and disease control is crucial. Simultaneously, training programs should continue to build the capacity of local personnel in diagnostics and surveillance. Ongoing research into more affordable, user-friendly diagnostic methods tailored to Bangladesh’s context should be encouraged (Fig. 9).

## Economic Impact and Future Prospective of Bovine Brucellosis

**Figure 10.**
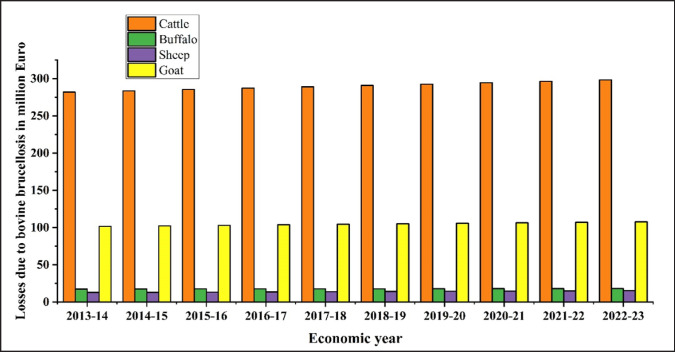
Financial losses (in Million Euro) associated with various species of ruminants due to bovine brucellosis from 2013-23.

A Bangladeshi economic impact study on bovine brucellosis suggested that the yearly economic impact resulting from bovine brucellosis among indigenous cows in Bangladesh is projected to reach Euro currency (€) 720,000, with an additional €12 per cross-bred cow [70]. We have considered that cows and buffaloes share similar sizes and disease prevalence characteristics, whereas goats and sheep are approximately one-fourth the size, with an estimated loss of €4 per year. In recent years, due to the bovine brucellosis, a consistent upward trend in financial losses was observed, measured in million Euro, for cattle (281.856 million–298.272 million), buffalo (17.484 million–18.192 million), sheep (12.824 million–15.308 million), and goats (101.756 million–107.78 million) over 10 years from 2013–14 to 2022–23 (Fig. 10).

**Figure 11.**
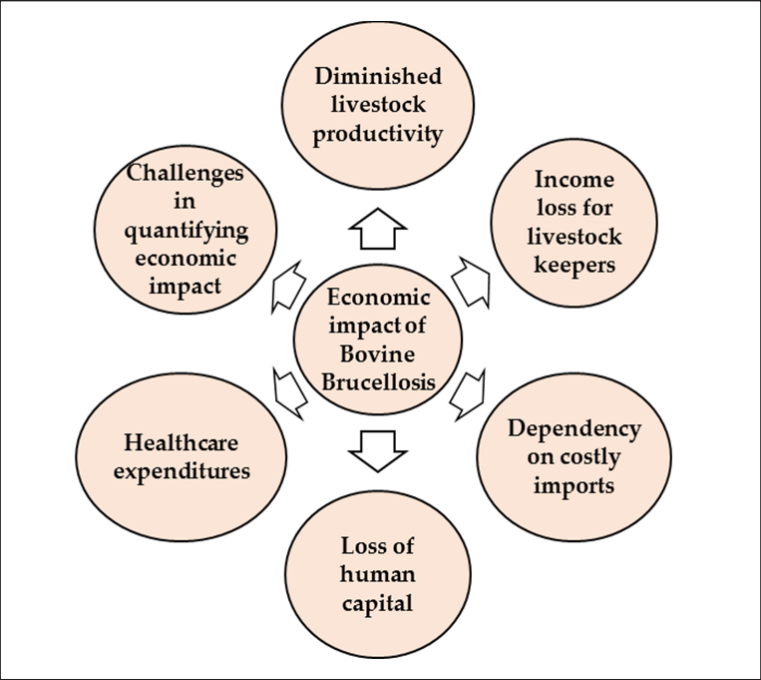
The economic impact of bovine brucellosis in Bangladesh.

One of the most palpable economic impacts of bovine brucellosis in Bangladesh is its detrimental effect on livestock productivity [10]. The disease primarily affects cattle, buffalo, and small ruminants, causing reproductive issues such as abortions, stillbirths, and reduced fertility rates [15]. These reproductive losses translate into decreased milk and meat production [71]. As a result, affected animals become less productive, leading to diminished incomes for livestock farmers (Fig. 11).

Bovine brucellosis-induced reductions in milk and meat production result in financial losses for farmers who rely on the sale of these products [72]. Moreover, the disease’s impact extends beyond the individual animal level; entire herds can become infected, exacerbating the economic strain on livestock keepers. The decline in domestic milk and meat production due to bovine brucellosis has broader implications for the nation’s economy. Bangladesh increasingly depends on costly imports to meet its demand for dairy and meat products. These imports strain the country’s foreign exchange reserves and contribute to trade imbalances [73]. The economic repercussions reverberate through various sectors, impacting the nation’s overall financial stability. Infected animals may require veterinary care, which can be costly for farmers. Additionally, if zoonotic transmission occurs, infected individuals require medical attention. Healthcare expenditures encompass diagnosis, treatment, and control measures, all of which impose financial burdens on both individuals and the healthcare system [13].

Farmers and dairy workers, who often have close contact with infected animals or consume tainted dairy and meat products, are at risk of contracting the disease. Human infections result in losses not only in terms of health and well-being but also in terms of human capital. Infected individuals may be unable to work, leading to reduced productivity and income [74]. Quantifying the precise economic impact of bovine brucellosis in Bangladesh is a complex task. The interconnectedness of agriculture, trade, healthcare, and livelihoods makes it challenging to isolate the disease’s specific contributions to economic losses [65]. Additionally, underreporting and undiagnosed cases of bovine brucellosis make it difficult to estimate the true extent of the problem.

Vaccination campaigns targeting replacement heifers aim to reduce the prevalence of bovine brucellosis and prevent new infections, thereby limiting the associated economic losses [75]. Improving the country’s diagnostic capacity helps promptly identify infected animals, allowing for their segregation and culling to prevent the further spread of the disease within herds [2]. Raising awareness among farmers about bovine brucellosis and its economic consequences encourages proactive measures such as testing and vaccination. Stricter trade policies regarding the importation of livestock and livestock products can protect domestic industries from the competition of cheaper, potentially contaminated imports [53]. Improved data collection and epidemiological studies can provide a more accurate assessment of the economic losses attributable to bovine brucellosis. This information is vital for policy formulation and resource allocation [23]. An integrated approach that combines vaccination, diagnostics, and public awareness campaigns can mitigate the economic impact of bovine brucellosis by reducing infection rates and improving productivity, thereby enhancing the country’s overall economic well-being [15]. Collaboration between the livestock sector, public health authorities, and policymakers is essential. A One Health approach that addresses both animal and human health aspects of bovine brucellosis can lead to more effective control measures, ultimately reducing economic losses [27]. Investment in research and innovation, including the development of cost-effective diagnostics and vaccines, can contribute to economic resilience in the face of bovine brucellosis [35].

## One Health Approach

**Figure 12.**
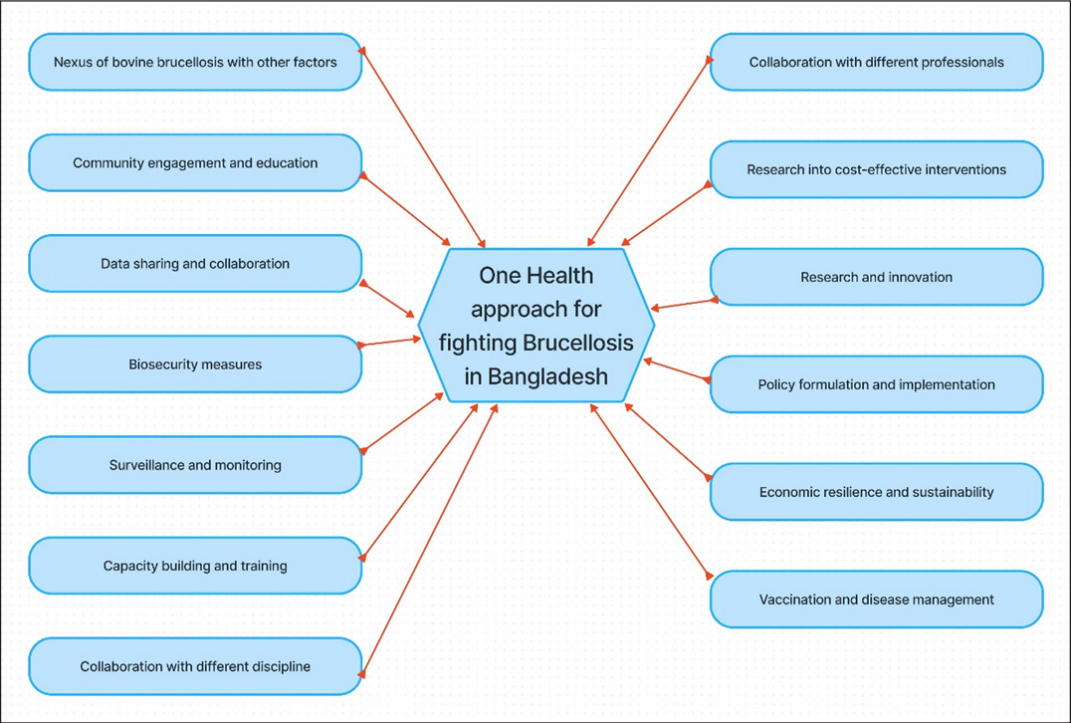
One Health approach for fighting Brucellosis in Bangladesh.

As the nation grapples with the complex and enduring issue of bovine brucellosis outbreaks, the application of a One Health approach emerges as a compelling strategy [27]. This comprehensive approach recognizes the interconnectedness of animal, human, and environmental health and seeks integrated solutions to address the multifaceted challenges posed by this disease [76]. The systematic One Health approach is presented in Figure 12.

In addressing the complex web of challenges posed by bovine brucellosis in Bangladesh, a One Health approach is imperative, as it transcends disciplinary boundaries and emphasizes collaborative efforts among veterinary professionals, public health authorities, policymakers, researchers, and communities [9]. Veterinary hospitals are responsible for diagnosing and treating infected animals, implementing vaccination programs, and monitoring disease prevalence [35]. Public health authorities, on the other hand, focus on human health surveillance, contact tracing, and educating at-risk populations [9]. By working together, these two sectors can create a more comprehensive understanding of bovine brucellosis transmission dynamics and its impact on both animals and humans.

Research and innovation are essential components of a One Health strategy, with collaborative research projects involving veterinarians, epidemiologists, microbiologists, and public health experts yielding valuable insights into disease prevalence, transmission routes, and risk factors [27]. Additionally, the development of cost-effective diagnostic tools, vaccines, and treatment regimens requires the synergy of veterinary and medical research [35]. In resource-limited Bangladesh, partnerships with international organizations and research institutions facilitate technology transfer, knowledge exchange, and funding access for critical research initiatives, accelerating the development of tailored, innovative solutions.

Effective policies are vital for One Health success, requiring policymakers to recognize the link between bovine brucellosis and diverse interests, with strict regulations on livestock movement, trade, and food safety essential for disease control [77]. Moreover, policies aimed at strengthening healthcare infrastructure, enhancing diagnostic capacity, and improving veterinary services are essential [27]. Harmonizing policies across sectors is a significant challenge but is crucial to ensuring a cohesive response. Interagency coordination and stakeholder engagement are key to aligning policies with the One Health approach. Community engagement and education are crucial for One Health, ensuring understanding of bovine brucellosis risks and promoting safe livestock handling, hygienic food practices, and vaccination [78]. Community-based initiatives empower individuals to implement biosecurity practices, seek timely veterinary care, and advocate for safe and hygienic dairy and meat products [79]. In urban areas, consumer awareness can drive market forces to favor products from disease-free sources.

Robust surveillance and monitoring are vital for tracking disease prevalence and identifying hotspots, with veterinarians and public health officials collaborating to share crucial data for effective disease control [15]. Early warning systems should be established to detect outbreaks promptly, allowing for swift intervention [80]. Vaccination campaigns are the cornerstone of bovine brucellosis control in livestock populations. Veterinarians play a central role in administering vaccines and monitoring their efficacy [40]. Research into cost-effective interventions is essential for resource-limited settings like Bangladesh, with collaborative studies identifying the most cost-efficient strategies for disease control and prevention [35]. These studies evaluate vaccine formulations, diagnostic tests, and treatment regimens to develop tailored, cost-effective solutions. Veterinarians advise on biosecurity practices, while public health authorities emphasize personal hygiene and safe food handling to reduce disease transmission in livestock herds [81].

Capacity building and training are essential components of a One Health approach. Veterinarians, medical professionals, and public health workers should receive training in zoonotic disease recognition and management [82]. This cross-training can enhance the capacity of healthcare systems to respond effectively to zoonotic disease threats. Data sharing and collaboration among stakeholders are vital for informed decision-making and coordinated responses. Establishing platforms for sharing epidemiological data, surveillance findings, and research outcomes can facilitate collaboration among veterinary and public health professionals [27]. International partnerships can further enhance data sharing and strengthen the country’s response to bovine brucellosis [83]. A One Health approach also emphasizes economic resilience and sustainability. By addressing the economic ramifications of bovine brucellosis, such as reduced livestock productivity and increased healthcare expenditures, Bangladesh can safeguard its economic stability. Innovative strategies, such as improving animal health and productivity through research and development, can reduce reliance on costly imports [84].

## Community Engagement and Awareness

### Understanding the significance of community engagement

Community engagement is an indispensable component of tackling zoonotic diseases like bovine brucellosis. In Bangladesh, where livestock farming is deeply ingrained in the culture and economy, involving local communities becomes even more crucial [10]. The multifaceted nature of this disease necessitates a collaborative approach that includes the active participation of farmers, livestock keepers, veterinarians, public health authorities, and consumers [85].

### Engaging farmers and livestock keepers

Farmers and livestock keepers are on the front lines of bovine brucellosis transmission. Engaging them in awareness campaigns and educational initiatives is vital for disease control [86]. Educating farmers about the clinical signs of bovine brucellosis in animals is crucial. This includes manifestations like abortion, infertility, and decreased milk production. Prompt recognition of these symptoms can lead to early intervention [6]. Farmers need to be aware of preventive measures, such as vaccination and biosecurity practices. Proper vaccination can significantly reduce the prevalence of the disease, and biosecurity measures can prevent its spread within and between herds [87]. Farmers should be encouraged to report suspected cases of bovine brucellosis to local veterinary authorities. Livestock trade is a potential avenue for disease transmission. Farmers should be educated about the risks associated with purchasing animals from unknown sources and the importance of buying from reputable dealers.

### Empowering veterinary professionals

Continuous training programs can update veterinarians on the latest diagnostic methods, treatment protocols, and prevention strategies related to bovine brucellosis [88]. Ensuring access to reliable diagnostic facilities and resources is critical. This enables veterinarians to accurately diagnose the disease and initiate appropriate measures [68]. Encouraging veterinarians to share disease-related data with relevant authorities contributes to a more comprehensive understanding of the disease’s prevalence and distribution [89].

### Public awareness and education

Beyond the agricultural sector, raising public awareness about bovine brucellosis is essential, as this disease poses zoonotic risks. Public awareness campaigns should focus on educating consumers about the risks of consuming raw or inadequately cooked dairy and meat products from infected animals, which can reduce the chances of human infection [47]. Informing the public about the symptoms of human Brucellosis, such as fever, joint pain, and fatigue, can lead to early medical intervention [90]. Promoting simple preventive measures, such as proper hand washing after handling animals or animal products, can reduce the risk of zoonotic transmission [91].

### Challenges in community engagement

Many farmers, especially in rural areas, lack access to educational materials and training opportunities. Limited resources can hinder their ability to adopt preventive practices [27]. Some traditional practices, such as consuming raw milk, may be deeply ingrained in the culture. Overcoming these practices requires culturally sensitive educational approaches [92]. Access to healthcare services for humans and animals is unevenly distributed in Bangladesh. This can hinder the prompt diagnosis and treatment of Brucellosis cases [93].

### Future perspectives in community engagement

Utilizing mobile technology for disseminating information can reach even remote communities. Short message service (SMS) alerts, voice messages, and mobile apps can provide timely updates and guidance [79]. Training and deploying community health workers who can educate communities about zoonotic diseases can be an effective approach [94]. Leveraging social media and online platforms for awareness campaigns can engage urban populations and the younger generation. By fostering partnerships, educating stakeholders, and overcoming challenges, Bangladesh can bolster its efforts to control and prevent the spread of this disease.

## Trends of Bovine Brucellosis Research in Bangladesh 

**Figure 13.**
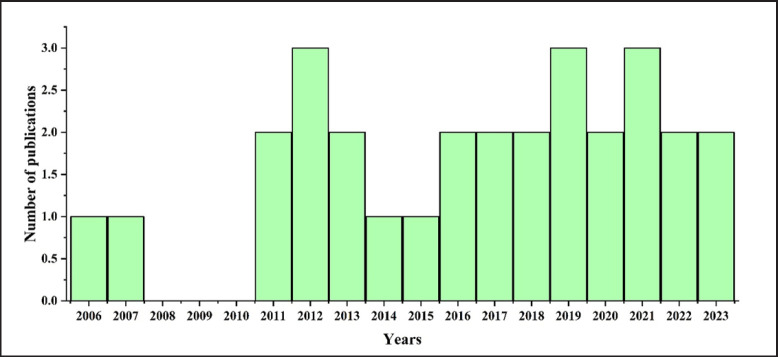
Research trend analysis of bovine brucellosis in Bangladesh.

A total of 29 publications since 2006 reported the facts and findings of bovine brucellosis from Bangladesh (Fig. 13). On the other hand, most of the research is concentrated in the Mymensingh region of the country due to the available research facilities in different universities in the area and most probably for the Bangladesh Agricultural University [10] (Supplementary Table 1 and Fig. 13).

In Bangladesh, brucellosis poses a multifaceted challenge, with various factors contributing to its prevalence and transmission dynamics. Direct contact with aborted fetuses, retained placenta, and vaginal fluid emerges as a key mode of transmission among cows [10]. Furthermore, consuming raw milk and dairy products heightens the risk of brucellosis transmission [47]. The prevalence of brucellosis in smallholder dairy farms in Bangladesh’s Mymensingh district underscores the pervasive nature of the disease within local agricultural communities. Also, the fact that bovine brucellosis is common in both urban and rural areas, like the Chittagong Metropolitan Area, shows how diseases can move from one to the other. To stop the disease from spreading to livestock, we need to use thorough screening methods [2].

Beyond brucellosis, Bangladesh grapples with a spectrum of zoonotic diseases, further complicating public health efforts. Major outbreaks of diseases like the Nipah virus and avian influenza underscore the interconnectedness of human and animal health, necessitating a holistic and One Health approach for effective disease management [7]. Also, finding common bacterial zoonoses like tuberculosis in dead cattle shows how complicated the food chain is for disease transmission, making it even more important to have strong surveillance systems and diagnostic tools [95]. In this situation, the discovery of *Brucella* abortus biovar 3 in dairy cow milk not only poses a health risk to humans, but it also shows how diseases can spread around the world, calling for countries to work together to create effective ways to control and stop them [21].

We determined the true prevalence rates for both cattle and goats, emphasizing the widespread nature of brucellosis within these populations. The focus on surveillance and control in sheep emphasizes the need for targeted interventions to curb disease transmission and mitigate its impact on animal health and production [96]. Additionally, the observed variability in brucellosis prevalence among small-scale cattle systems and selected farms in Bangladesh highlights the nuanced challenges in disease management, necessitating tailored approaches to address specific risk factors and transmission pathways. Despite the relatively low prevalence rates reported in goats and sheep, brucellosis is present in multiple species, including livestock and domesticated animals. Also, the results of changes in the blood of *Brucella* abortus antibody-positive cows and the confirmed seroprevalence among high-risk occupational groups give us important information about how brucellosis shows up in Bangladesh and how it spreads. Particularly in regions like Mymensingh and Bogra districts, where the disease significantly hampers livestock development in goats and sheep, understanding the epidemiological dynamics is essential for designing effective interventions [25]. Studies highlight the association between specific clinical histories in goats and brucellosis. Furthermore, research reveals that pregnancy has no significant impact on the prevalence of brucellosis in cows.

**Figure 14.**
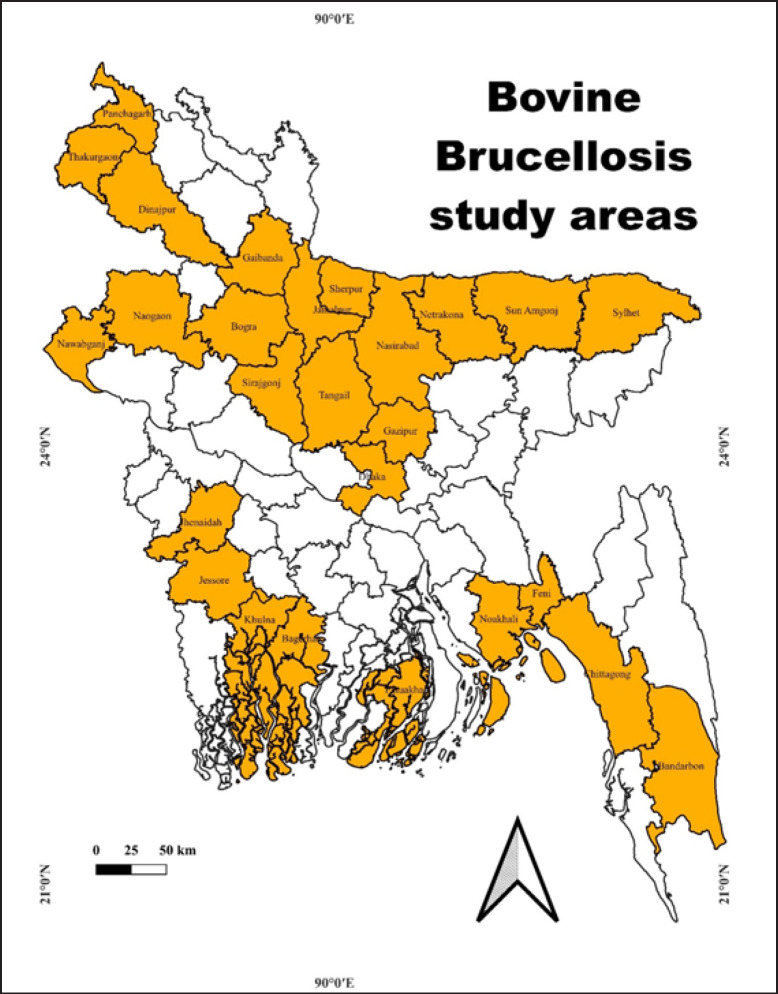
The prevalence, distribution, and research on bovine brucellosis are majorly concentrated in the northern part of Bangladesh.

Addressing the complex landscape of zoonotic diseases in Bangladesh requires a multifaceted approach encompassing veterinary public health (VPH), community engagement, and targeted control measures. Establishing VPH units, enhancing disease surveillance capabilities, and fostering collaboration between veterinary and health departments are paramount for effective disease control. Furthermore, the recommendation to employ multiple diagnostic tests for brucellosis diagnosis underscores the importance of a nuanced, evidence-based approach tailored to the local context [18]. Ultimately, mitigating the impact of zoonotic diseases like brucellosis in Bangladesh necessitates a concerted effort at the intersection of human, animal, and environmental health, guided by scientific research, policy support, and community participation.

We discovered that bovine brucellosis prevalence is present in 26 districts, and on the other hand, most of the research related to bovine brucellosis was conducted in the northern part of Bangladesh (Fig. 14) due to the availability of grazing land, farming facilities, and other amenities [10].

## Conclusion

In Bangladesh’s complex livestock and public health landscape, bovine brucellosis has posed a persistent threat for decades. Our comprehensive review provides insights into its historical evolution, current impact, and future challenges. From its unnoticed presence pre-independence to its economic and public health ramifications today, the disease remains a formidable adversary. Addressing diagnostic limitations, economic strain, and public health risks requires a multidisciplinary approach involving stakeholders from the livestock industry, public health, and policymaking. By crafting sustainable strategies tailored to Bangladesh’s context, we can envision a future free from the shadow of bovine brucellosis, ensuring the health, well-being, and economic stability of the nation.

## Figures and Tables

**Supplementary Table 1.** Output and the trends of bovine Brucellosis researches in Bangladesh.

Year	Study area	Key findings	Animal	Ref.
2023	Sylhet	Direct contact with aborted fetuses, retained placenta, and vaginal fluid is a key source of brucellosis transmission in cows. Factors such as repeat breeding, abortion, parity, and reproductive issues increase the risk. These findings aid surveillance and control efforts. Further research is needed to explore brucellosis epidemiology in Sylhet, including transmission dynamics, environmental factors, geography, and economic impact.	Cow	[10]
2023	Multiple districts; Review	Consuming raw milk and dairy products can transmit brucellosis, a significant global health concern. *Brucella* is found in various milk sources, posing a risk from cow, buffalo, camel, and goat milk, as well as cheese. Unpasteurized milk is a leading cause of human brucellosis, with infection rates ranging from 33.9% to 100%. Detection of *Brucella* antibodies in milk can be done through tests like the milk ring test and ELISA, while real-time PCR quickly identifies *Brucella* organisms. Prevention measures include pasteurization, testing with MRT, immunization, and increased public awareness.	Multiple	[47]
2022	Mymensingh	The study identified prevalent bacterial zoonotic diseases, including tuberculosis, leptospirosis, and listeriosis, in slaughtered cattle. Tuberculosis was the most widespread, followed by leptospirosis and listeriosis. Brucellosis wasn't found, likely due to the limited sample size. The high prevalence of tuberculosis in slaughtered cattle raises health concerns for both humans and animals through the food chain. The study highlights the importance of sensitive molecular diagnostic tools and epidemiological surveys to detect, prevent, and control these diseases effectively at slaughterhouses.	-	[95]
2022	Mymensingh	Brucellosis prevalence in smallholder dairy farms in Bangladesh's Mymensingh district is 3.9% using serological tests. Cows with repeat breeding or retention of fetal membranes have significantly higher odds of infection. This disease affects animal health and poses risks to humans through unpasteurized milk. Serological tests are crucial for monitoring and controlling brucellosis, reducing economic losses, and mitigating human health risks.	Cow	[49]
2021	Chittagong	In the Chittagong Metropolitan Area of Bangladesh, bovine brucellosis has a seroprevalence of 21.5% (RBPT) and 7.6% (cELISA). Cows with reproductive disorders and low milk production have higher seropositivity odds. About 7.6% of cattle have acute brucellosis, and 1.3% have chronic brucellosis. Screening should prioritize these cows to control the disease and minimize economic losses.	Cow	[2]
2021	Bangladesh	This paper provides a review of major zoonotic diseases in Bangladesh and their associated risk factors. It highlights recurring outbreaks of Nipah virus, *Bacillus anthracis*, and avian influenza (H5N1) transmission to humans. The close interaction between people and animals in Bangladesh raises zoonotic disease risks, emphasizing the need for a one-health approach to address this public health threat. The review is based on data extracted from over 150 relevant publications.	Multiple	[7]
2021	Multiple districts	In selected areas of Bangladesh, Brucellosis prevalence in goats was 4.33% via RBPT and 2.40% using c-ELISA, with higher rates in does aged 3–4 years. The overall prevalence was relatively low. Further bacteriological investigations are needed to confirm the presence of *Brucella* organisms in Bangladeshi goats. Increased scientific work and surveillance are crucial to combat zoonotic diseases like Brucellosis in the livestock industry.	Buck and doe	[64]
2020	Multiple districts	The study in Bangladesh found a 1.5% seroprevalence of *Brucella* in dairy cattle using the sensitive iELISA technique. It emphasized the zoonotic significance of Brucellosis, causing reproductive losses like late abortions, weak calves, stillbirths, and infertility in mature animals, especially in farms with recent abortion history	Cow	[97]
2020	Sylhet	High-risk groups in Bangladesh have a higher sero-prevalence of human brucellosis due to close animal contact, especially among dairy farm workers. Since there's no human brucellosis vaccine, animal transmission remains a significant concern. The economic impact of brucellosis in Bangladesh needs evaluation. To combat this zoonotic disease, measures like awareness campaigns, vaccination, and cross-border screening are crucial to prevent both animal and human transmission.	Human	[5]
2019	Multiple districts	This study identified *Brucella abortus* biovar 3 in dairy cattle milk in Bangladesh, posing a public health risk due to unpasteurized milk consumption. The isolates are genetically related to those in Brazil and China, suggesting potential epidemiological links. Further analysis like whole genome sequencing is needed for a deeper understanding. MLVA-16 profiles align with those from Brazil in the international database, indicating a global connection.	Cow	[21]
2019	Bangladesh	Veterinary Public Health (VPH) is gaining importance in Bangladesh due to changes in animal production. Constraints include a lack of VPH services at all levels, budget shortages, and limited qualified personnel and lab facilities. Priorities include establishing VPH units, robust disease surveillance, a legal framework, public health awareness, and collaboration with health departments. Veterinary expertise at the policy level is crucial, along with strengthening VPH with better labs and trained personnel.	-	[96]
2019	Mymensingh	The study recommends using both iELISA and SAT tests for diagnosing bovine brucellosis in Bangladesh. In the Mymensingh district, the true prevalence was 0.6%, while a government-owned dairy farm had a prevalence of 20.4%. Removing infected cattle is recommended to control the disease. Factors like herd size and irregular testing may explain the higher prevalence on the dairy farm. The study employed a Bayesian latent class model to account for test dependencies and performed sensitivity analysis with different prior information sets.	Cow	[18]
2018	Mymensingh	Brucellosis has a significant economic impact in Mymensingh district, Bangladesh, with a 1.6% prevalence in goats and sheep. The annual financial loss is approximately 605,455 US dollars, emphasizing the need for better prevention and control strategies, including accurate diagnosis and vaccination programs.	Goat and sheep	[65]
2018	Multiple districts	The study from Bangladesh found a 6.6% seroprevalence of brucellosis in cattle, with higher rates in cases of abortion and infertility. *Brucella* spp. were successfully isolated from seronegative dairy cows with specific clinical histories. The study highlights the importance of isolating *Brucella* for effective control measures and recommends using bacteriological and PCR tests alongside serological tests for detection and eradication in Bangladesh.	Cow	[44]
2017	Multiple districts	*Brucella abortus*, not *Brucella melitensis*, was detected in both humans and domestic ruminants in Bangladesh, with milk as a potential source of infection. The study confirms *B. abortus* prevalence but suggests that brucellosis may not be the primary cause of abortion in domestic ruminants. Limitations included a small sample size, non-random sampling, and the inability to isolate *Brucella* from seropositive humans due to facility constraints. Further investigation is needed to understand the presence of* B. melitensis* in Bangladesh, especially in goat foetal membranes from different regions.	Multiple	[33]
2017	Dinajpur and Mymensingh	The study found a true prevalence of 9.70% for cattle and 6.3% for goats in Bangladesh. SAT was sensitive (69.6%–78.9%), and iELISA was specific (97.4%–98.8%). Factors like natural breeding in cattle and exotic goat breeds were linked to higher seroprevalence. No single test was recommended for diagnosis alone. Bayesian analysis and convergence diagnostics were employed. Multivariate logistic regression identified potential risk factors for brucellosis at the animal level.	Cattle and goat	[98]
2016	Mymensingh	In Bangladesh, due to the low prevalence of human brucellosis, relying on a single serological test for routine diagnosis is not recommended. Instead, employing a second test with high specificity or targeting individuals with known risk factors or clinical symptoms can enhance the accuracy of diagnosis.	Human	[99]
2016	Multiple districts	In Bangladesh, a 5% seroprevalence of ovine brucellosis in sheep was found. Higher rates were seen in certain districts, with adult and female sheep more affected. The study emphasizes nationwide surveillance and recommends culling of seropositive sheep for control. Further research for *Brucella* characterization is needed.	Sheep	[100]
2015	Multiple districts	Brucellosis prevalence in small-scale cattle and subsistence systems is very low and naturally controlled. No further control programs are recommended due to poor socioeconomic conditions. However, in central cattle breeding and dairy farms, where prevalence is very high, immediate control measures like calf hood vaccination are advised. Combining SAT and iELISA tests helps determine infection stage in high-prevalence scenarios. Brucellosis prevalence in goats and sheep is low, around 1, requiring cautious interpretation due to lower positive predictive value.	Multiple	[101]
2014	Dhaka	In selected farms in Bangladesh, a study found a low prevalence of brucellosis in dairy cattle, with 4.20% positive in the Rose-Bengal test and 1.20% confirmed by I-ELISA. It was more prevalent in females, with associations to abortion and age. The study suggests a test-and-slaughter eradication program is feasible due to the low prevalence. I-ELISA, with higher specificity, contributed to the lower prevalence determination. Contact with wild animals and dogs were mentioned as potential factors affecting prevalence.	Cow	[102]
2013	Mymensingh	The study found brucellosis prevalence in goats and sheep in Bangladesh to be 1% and 1.2%, respectively. Sensitivity and specificity were high for the indirect ELISA (iELISA) test, Rose Bengal Test (RBT), and Slow Agglutination Test (SAT). Conditional dependence was observed between these tests, suggesting a combined approach for accurate diagnosis in small ruminants.	Goat and sheep	[103]
2013	Review; Bangladesh	Brucellosis exists in livestock, domesticated animals, and humans in surveyed regions of Bangladesh. Seroprevalence variations relate to ecological factors, livestock density, practices, and test methods. Female animals are more susceptible. Prevalence varies in farm animals due to management, population dynamics, and biological factors. Mature, pregnant animals and those with abortion history are at higher risk, posing transmission risks to others.	Multiple	[104]
2012	Chittagong	Hematological values in *Brucella abortus* antibody-positive cows showed varied changes, including lower Hb and MCHC but higher MCV, neutrophils, monocytes, and eosinophils. Other values remained within reference ranges, with no statistical significance. Further research is needed to understand potential alterations at different levels due to *Brucella*.	Cow	[105]
2012	Multiple districts	In the Dhaka division of Bangladesh, the seroprevalence of brucellosis in a high-risk occupational group (HROG) was 4.4%, confirmed by real-time PCR. Livestock farmers in positive herds had a higher seropositivity risk, with goat contact as a significant transmission factor. Variability in cases was observed within districts, linked to the duration of animal contact. Data collection involved face-to-face interviews and ethical clearances. The study included various occupational groups in Mymensingh, Sherpur, and Dhaka districts.	Multiple	[106]
2012	Chittagong	Bovine brucellosis was widespread in Chittagong, Bangladesh, with higher prevalence on commercial farms. The study urged comprehensive brucellosis management in all farms, especially in Chittagong, and called for further research to identify species, transmission dynamics, and safer management practices.	Cow	[107]
2011	Multiple districts	Brucellosis prevalence in Bangladesh's ruminants varies: 2.87% in buffaloes, 2.66% in cattle, 3.15% in goats, and 2.31% in sheep. The study reveals links between brucellosis, abortion, and age, with females having a higher prevalence in cattle, goats, and sheep, while buffaloes show insignificantly higher rates in males. This data provides a baseline for future studies and control measures, potentially due to infections within the female reproductive tract.	Multiple	[63]
2011	Bogra and Mymensingh	In Bangladesh's Mymensingh and Bogra districts, brucellosis was found in 2.50% of goats and 1.25% of sheep, primarily in females, with a link to abortion. This zoonotic disease hampers livestock development and health but often goes undiagnosed, impacting sheep and goat reproduction. Brucellosis, including *Brucella melitensis*, remains a global concern, likely underreported in its prevalence.	Goat and sheep	[25]
2007	Multiple districts	In Bangladesh, a study using serological tests found that 2.3% of goats had brucellosis, with higher prevalence in aborted goats (20%), those with placental retention (13.3%), and those with abnormal uterine discharge (10%).	Goat	[108]
2006	Mymensingh and Sherpur	Pregnancy doesn't significantly affect brucellosis prevalence in cows. Screening tests like RBT and PAT were used, with TAT for confirmation. Organized farms had a 75% prevalence by RBT and PAT but only 5% by TAT. For rural cows, it was 30.8% by RBT and PAT and 19.2% by TAT, with no significant difference. Transmission on organized farms may involve introducing infected cows and potential spread by dairy attendants.	Cow	[109]

## List of abbreviations

CFT, complement fixation test; DNA, deoxyribonucleic acid; ELISA, enzyme-linked immunosorbent assay; €, Euro currency; Ig, Immunoglobulin; iELISA, indirect enzyme-linked immunosorbent assay; MRT, milk ring test; PCR, polymerase chain reaction; RBPT, rose Bengal plate test; SAT, slow agglutination test; STAT, serum tube agglutination test; SMS, short message service; VPH, veterinary public health.

## References

[1] Holt HR, Bedi JS, Kaur P, Mangtani P, Sharma NS, Gill JPS (2021). Epidemiology of brucellosis in cattle and dairy farmers of rural Ludhiana, Punjab. PLoS Neglect Trop Dis.

[2] Islam S, Barua SR, Moni SP, Islam A, Rahman AKMA, Chowdhury S (2021). Seroprevalence and risk factors for bovine brucellosis in the Chittagong Metropolitan Area of Bangladesh. Vet Med Sci.

[3] Mustafa AHM (1984). *Brucella* antibodies in the sera of domestic livestock in Bangladesh. Trop Anim Health Prod.

[4] Amin KMR, Rahman MB, Rahman MS,  Han JC, Park JH, Chae JS (2005). Prevalence of *Brucella *antibodies in sera of cows in Bangladesh. J Vet Sci.

[5] Akhtar J, Chowdhury OA, Das P, Sinha SP (2020). Seroprevalence of human brucellosis among high risk and normal individuals of Sylhet district in Bangladesh. Bangladesh Med Res Coun Bull.

[6] Khan MZ, Zahoor M (2018). An overview of brucellosis in cattle and humans, and its serological and molecular diagnosis in control strategies. Trop Med Inf Dis.

[7] Chowdhury S, Aleem MA, Khan MSI, Hossain ME, Ghosh S, Rahman MZ (2021). Major zoonotic diseases of public health importance in Bangladesh. Vet Med Sci.

[8] Sultana N, Pervin M, Sultana S, Islam M, Mostaree M, Khan MAHNA (2022). Pathological study and molecular detection of zoonotic diseases in small ruminants at slaughterhouses in Mymensingh, Bangladesh. Vet World.

[9] Tulu D (2022). Bovine Brucellosis: epidemiology, public health implications, and status of Brucellosis in Ethiopia. Vet Med Res Rep.

[10] Deb Nath N, Ahmed SSU, Malakar V, Hussain T, Chandra Deb L, Paul S (2023). Sero-prevalence and risk factors associated with brucellosis in dairy cattle of Sylhet district, Bangladesh: a cross-sectional study. Vet Med Sci.

[11] Efrem GH, Mihreteab B, Ghebremariam MK, Okbamichael T, Ghebresilasie Y, Mor SM (2023). Prevalence of brucellosis and associated risk factors in dairy cattle in Maekel and Debub regions, Eritrea. Front Vet Sci.

[12] Warioba JP, Karimuribo ED, Komba EVG, Kabululu ML, Minga GA, Nonga HE (2023). Occurrence and risk factors of Brucellosis in commercial cattle farms from selected districts of the eastern coast zone, Tanzania. Vet Med Int.

[13] Franc KA, Krecek RC, Häsler BN, Arenas-Gamboa AM (2018). Brucellosis remains a neglected disease in the developing world: a call for interdisciplinary action. BMC Public Health.

[14] Godfroid J (2017). Brucellosis in livestock and wildlife: zoonotic diseases without pandemic potential in need of innovative one health approaches. Arc Public Health.

[15] Khurana SK, Sehrawat A, Tiwari R, Prasad M, Gulati B, Shabbir MZ (2021). Bovine brucellosis–a comprehensive review. Vet Quartly.

[16] Harzing A (2007). Publish or Perish. https://harzing.com/resources/publish-or-perish.

[17] Rahman MA, Mia AS (1970). A study of brucellosis in Bangladesh. Bangladesh J Anim Sci.

[18] MoFL (2023). Ministry of Fisheries & Livestock, Government of the People’s Republic of Bangladesh. MoFL.

[19] Rockström J, Williams J, Daily G, Noble A, Matthews N, Gordon L (2017). Sustainable intensification of agriculture for human prosperity and global sustainability. Ambio.

[20] Moller K, Eeswaran R, Nejadhashemi AP, Hernandez-Suarez JS (2023). Livestock and aquaculture farming in Bangladesh: current and future challenges and opportunities. Cogent Food Agric.

[21] Islam MS, Garofolo G, Sacchini L, Dainty AC, Khatun MM, Saha S (2019). First isolation, identification and genetic characterization of *Brucella abortus biovar* 3 from dairy cattle in Bangladesh. Vet Med Sci.

[22] Deka RP, Magnusson U, Grace D, Lindahl J (2018). Bovine brucellosis: prevalence, risk factors, economic cost and control options with particular reference to India- a review. Infect Ecol Epidemiol.

[23] Grout L, Baker MG, French N, Hales S (2020). A review of potential public health impacts associated with the global dairy sector. GeoHealth.

[24] Rahman MS, Ali Hahsin MF, Ahasan MS, Her M, Kim JY, Kang S Il (2011). Brucellosis in sheep and goat of Bogra and Mymensingh districts of Bangladesh. Korean J Vet Res.

[25] Deneke TT, Bekele A, Moore HL, Mamo T, Almaw G, Mekonnen GA (2022). Milk and meat consumption patterns and the potential risk of zoonotic disease transmission among urban and peri-urban dairy farmers in Ethiopia. BMC Public Health.

[26] Moriyón I, Blasco JM, Letesson JJ, De Massis F, Moreno E (2023). Brucellosis and one health: inherited and future challenges. Microorganisms.

[27] Hasan MK, Kumar L (2022). Changes in coastal farming systems in a changing climate in Bangladesh. Reg Environ Change.

[28] Chowdhury T, Ahmed J, Hossain MT, Roy MC, Ashik-Uz-Zaman M, Uddin MN (2023). Knowledge, attitudes and biosecurity practices among the small-scale dairy farmers in Sylhet district, Bangladesh. Vet Med Sci.

[29] Ngoshe YB, Etter E, Gomez-Vazquez JP, Thompson PN (2023). Knowledge, attitudes, and practices of communal livestock farmers regarding animal health and zoonoses in far northern KwaZulu-Natal, South Africa. Int J Environ Res Public Health.

[30] Garrido-Haro A, Barrionuevo-Samaniego M, Moreno-Caballeros P, Burbano-Enriquez A, Sánchez-Vázquez MJ, Pompei J (2023). Seroprevalence and risk factors related to bovine Brucellosis in continental Ecuador. Pathogens.

[31] Osmani A, Robertson ID, Habib I (2021). Seroprevalence and risk factors for foot-and-mouth disease in cattle in Baghlan Province, Afghanistan. Vet Med Sci.

[32] Rahman AKMA, Saegerman C, Berkvens D, Melzer F, Neubauer H, Fretin D (2017). *Brucella abortus* is prevalent in both humans and animals in Bangladesh. Zoon Public Health.

[33] Elbehiry A, Aldubaib M, Marzouk E, Abalkhail A, Almuzaini AM, Rawway M (2023). The development of diagnostic and vaccine strategies for early detection and control of human Brucellosis, particularly in endemic areas. Vaccines.

[34] Waringa NMA, Waiboci LW, Bebora L, Kinyanjui PW, Kosgei P, Kiambi S (2023). Human brucellosis in Baringo County, Kenya: evaluating the diagnostic kits used and identifying infecting *Brucella* species. PLoS One.

[35] Matope G, Gadaga MB, Bhebhe B, Tshabalala PT, Makaya PV (2023). Bovine brucellosis and tuberculosis at a livestock–wildlife interface in Zimbabwe: a nexus for amplification of a zoonosis or a myth?. Vet Med Sci.

[36] Win TTZ, Campbell A, Magalhaes SRJ, Oo KN, Henning J (2023). Perceptions of livestock value chain actors (VCAs) on the risk of acquiring zoonotic diseases from their livestock in the central dry zone of Myanmar. BMC Public Health.

[37] Penrith ML, van Heerden J, Pfeiffer DU, Oļševskis E, Depner K, Chenais E (2023). Innovative research offers new hope for managing African swine fever better in resource-limited smallholder farming settings: a timely update. Pathogens.

[38] Heidary M, Dashtbin S, Ghanavati R, Mahdizade Ari M, Bostanghadiri N, Darbandi A (2022). Evaluation of Brucellosis vaccines: a comprehensive review. Front Vet Sci.

[39] Chacón-Díaz C, Zabalza-Baranguá A, Román BS, Blasco JM, Iriarte M, Salas-Alfaro D (2021). *Brucella abortus* S19 GFP-tagged vaccine allows the serological identification of vaccinated cattle. PLoS One.

[40] De Massis F, Sacchini F, D’Alterio N, Migliorati G, Ferri N, Rossi E (2023). *Brucella abortus* strain RB51 administered to prepubescent water buffaloes, from vaccination to lactation: Kinetics of antibody response and vaccine safety. Microorganisms.

[41] Khatibi M, Abdulaliyev G, Azimov A, Ismailova R, Ibrahimov S, Shikhiyev M (2021). Working towards development of a sustainable brucellosis control programme, the Azerbaijan example. Res Vet Sci.

[42] Islam MS, Islam MA, Khatun MM, Saha S, Basir MS, Hasan MM (2018). Molecular detection of *Brucella* spp. from milk of seronegative cows from some selected area in Bangladesh. J Pathogens.

[43] Legesse A, Mekuriaw A, Gelaye E, Abayneh T, Getachew B, Weldemedhin W (2023). Comparative evaluation of RBPT, I-ELISA, and CFT for the diagnosis of brucellosis and PCR detection of *Brucella* species from Ethiopian sheep, goats, and cattle sera. BMC Microbiol.

[44] Nandini P, Jakka P, Murugan S, Mazumdar V, Kumar D, Prakash R (2023). Immuno-profiling of *Brucella* proteins for developing improved vaccines and DIVA capable serodiagnostic assays for brucellosis. Front Microbiol.

[45] Islam MS, Islam MA, Rahman MM, Islam K, Islam MM, Kamal MM (2023). Presence of *Brucella* spp. in milk and dairy products: a comprehensive review and its perspectives. J Food Quality.

[46] Rahman AKMA, Smit S, Devleesschauwer B, Kostoulas P, Abatih E, Saegerman C (2019). Bayesian evaluation of three serological tests for the diagnosis of bovine brucellosis in Bangladesh. Epidemiol Infect.

[47] Hota A, Maiti SK, Ashmi M, Doimari S, Kumar B, Singh DK (2021). Seroprevalence and associated risk factors of bovine Brucellosis in Chhattisgarh Plains, India. Indian J Anim Res.

[48] Tithy NS, Islam SMS, Hussaini SMAK, Sharmy ST, Maruf A, Yeasmin F (2022). Prevalence and associated risk factors of bovine Brucellosis in smallholder dairy cows of Mymensingh district in Bangladesh. J Vet Med One Health Res.

[49] Qiu Y, Guitian J, Webster JP, Musallam I, Haider N, Drewe JA (2023). Global prioritization of endemic zoonotic diseases for conducting surveillance in domestic animals to protect public health. Phil Tran Royal Soc B Biol Sci.

[50] Saidu AS, Singh M, Kumar A, Mahajan NK, Mittal D, Chhabra R (2022). Studies on intra-ocular vaccination of adult cattle with reduced dose *Brucella* abortus strain-19 vaccine. Heliyon.

[51] Lokamar PN, Kutwah MA, Atieli H, Gumo S, Ouma C (2020). Socio-economic impacts of brucellosis on livestock production and reproduction performance in Koibatek and Marigat regions, Baringo County, Kenya. BMC Vet Res.

[52] Dadar M, Tiwari R, Sharun K, Dhama K (2021). Importance of brucellosis control programs of livestock on the improvement of one health. Vet Quart.

[53] Galińska EM, Zagórski J, Em G, Brucellosis ZJ (2013). Brucellosis in humans-etiology, diagnostics, clinical forms. Ann Agri Environ Med.

[54] Singh BB, Khatkar MS, Aulakh RS, Gill JPS, Dhand NK (2018). Estimation of the health and economic burden of human brucellosis in India. Prev Vet Med.

[55] Owusu-Kwarteng J, Akabanda F, Agyei D, Jespersen L (2020). Microbial safety of milk production and fermented dairy products in Africa. Microorganisms.

[56] Béjaoui A, Ben Abdallah I, Maaroufi A (2022). *Brucella* spp. contamination in artisanal unpasteurized dairy products: an emerging foodborne threat in Tunisia. Foods.

[57] Sarolidou G, Axelsson J, Kimball BA, Sundelin T, Regenbogen C, Lundström JN (2020). People expressing olfactory and visual cues of disease are less liked. Phil Tran Royal Soc B Biol Sci.

[58] Nyokabi NS, Lindahl JF, Phelan LT, Berg S, Gemechu G, Mihret A (2023). Exploring the composition and structure of milk and meat value chains, food safety risks and governance in the Addis Ababa and Oromia regions of Ethiopia. Front Sust Food System.

[59] Fagnani R, Nero LA, Rosolem CP (2021). Why knowledge is the best way to reduce the risks associated with raw milk and raw milk products. J Dairy Res.

[60] Stemshorn BW, Forbes LB, Eaglesome MD, Nielsen KH, Robertson FJ, Samagh BS (1985). A comparison of standard serological tests for the diagnosis of bovine brucellosis in Canada. Canadian J Comp Med.

[61] Ararsa G, Hirpa E, Amante M (2021). Complement fixation test for specific antibody detection against bovine Brucellosis in selected peasant sssociation of Guto Gida district, East Wollega Zone, Oromia, Ethiopia. Vet Med Int.

[62] Rahman MS, Faruk MO, Her M, Kim JY, Kang SI, Jung SC (2011). Prevalence of brucellosis in ruminants in Bangladesh. Vet Med.

[63] Al-Afifi AH, Almashhadany DA, Al-Azazi ASH, Khalaf AM, Odhah MNA, Al-Gabri NA (2022). Prevalence of *Brucella* spp. in milk from aborted and non-aborted animals in Dhamar governorate, Yemen. Italian J Food Safe.

[64] Nielsen K, Yu WL (2010). Serological diagnosis of brucellosis. Prilozi.

[65] Godfroid J, Nielsen K, Saegerman C (2010). Diagnosis of brucellosis in livestock and wildlife. Croatian Med J.

[66] Gwida M, El-Ashker M, Melzer F, El-Diasty M, El-Beskawy M, Neubauer H (2016). Use of serology and real time PCR to control an outbreak of bovine brucellosis at a dairy cattle farm in the Nile delta region, Egypt. Irish Vet J.

[67] Saxena HM, Chothe S, Kaur P (2015). Simple solutions to false results with plate/slide agglutination tests in diagnosis of infectious diseases of man and animals. MethodsX.

[68] Islam A, Hague M, Rahman A, Rahman M, Rahman A, Haque F (1983). Economic losses due to brucellosis among cattle in Bangladesh. Bangladesh Vet J.

[69] Calistri P, Iannetti S, Atzeni M, Di Bella C, Schembri P, Giovannini A (2013). Risk factors for the persistence of bovine brucellosis in Sicily from 2008 to 2010. Prev Vet Med.

[70] Lokamar PN, Kutwah MA, Munde EO, Oloo D, Atieli H, Gumo S (2022). Prevalence of brucellosis in livestock keepers and domestic ruminants in Baringo county, Kenya. PLoS Glob Public Health.

[71] Hénaux V, Calavas D (2017). Evaluation of the cost-effectiveness of bovine brucellosis surveillance in a disease-free country using stochastic scenario tree modelling. PLoS One.

[72] Ibarra M, Campos M, Ibarra C, Gladys U, Huera D, Gutiérrez M (2023). Financial losses associated with bovine Brucellosis (*Brucella abortus*) in Carchi-Ecuador. Open J Anim Sci.

[73] Ahmed BS, Osmani MG, Rahman AKMA, Hasan MM, Maruf AA, Karim MF (2019). Economic impact of caprine and ovine brucellosis in Mymensingh district, Bangladesh. Bangladesh J Vet Med.

[74] Yeasmin F, Maruf AA, Karim MF, Tasnin S, Rahman AKMA, Hasan MM (2019). Immune response of a heat killed *Brucella abortus* vaccine in guinea pig. Bangladesh J Vet Med.

[75] Mackenzie JS, Jeggo M (2019). The one health approach-why is it so important?. Trop Med Infect Dis.

[76] Behzadifar M, Shahabi S, Zeinali M, Ghanbari MK, Martini M, Bragazzi NL (2021). A policy analysis of agenda-setting of Brucellosis in Iran using a multiple-stream framework: health policy and historical implications. J Prev Med Hyg.

[77] Madzingira O, Byaruhanga C, Fasina FO, van Heerden H (2023). Assessment of knowledge, attitudes and practices relating to brucellosis among cattle farmers, meat handlers and medical professionals in Namibia. Vet Med Sci.

[78] Asakura S, Makingi G, John K, Kazwala R, Makita K (2022). Use of a participatory method for community-based Brucellosis control design in agro-pastoral areas in Tanzania. Front Vet Sci.

[79] Carpenter TE, Chrièl M, Greiner M (2007). An analysis of an early-warning system to reduce abortions in dairy cattle in Denmark incorporating both financial and epidemiologic aspects. Prev Vet Med.

[80] Dhand NK, Singh J, Josan HS, Singh BB, Jaswal N, Tiwari HK (2021). The feasibility and acceptability of various bovine brucellosis control strategies in India. Prev Vet Med.

[81] Tukana AS (2018). A study of Brucellosis in cattle within the Pacific Island community as a model for disease surveillance and reporting.

[82] Bansal D, Jaffrey S, Al-Emadi NA, Hassan M, Islam MM, Al-Baker WAA (2023). A new one health framework in Qatar for future emerging and re-emerging zoonotic diseases preparedness and response. One Health.

[83] Holt HR, Walker M, Beauvais W, Kaur P, Bedi JS, Mangtani P (2023). Modelling the control of bovine brucellosis in India. J Royal Soc Int.

[84] Ghanbari MK, Gorji HA, Behzadifar M, Sanee N, Mehedi N, Bragazzi NL (2020). One health approach to tackle brucellosis: a systematic review. Trop Med Health.

[85] Vinueza RL, Durand B, Ortega F, Salas F, Ferreira Vicente A, Freddi L (2023). Farm prevalence of bovine Brucellosis, farmer awareness, and local practices in small- and medium-scale cattle farms in a tropical region of Ecuador. Transbound Emerg Dis.

[86] Banai M, Jiang H, Peng X, Feng Y, Jiang H, Ding J (2021). The prevention and control of domesticated animal brucellosis. Biosafe Health.

[87] Tiwari HK, Proch V, Singh BB, Schemann K, Ward M, Singh J (2022). Brucellosis in India: comparing exposure amongst veterinarians, para-veterinarians and animal handlers. One Health.

[88] Bronner A, Morignat E, Calavas D (2015). Respective influence of veterinarians and local institutional stakeholders on the event-driven surveillance system for bovine brucellosis in France. BMC Vet Res.

[89] Jiang W, Chen J, Li Q, Jiang L, Huang Y, Lan Y (2019). Epidemiological characteristics, clinical manifestations and laboratory findings in 850 patients with brucellosis in Heilongjiang Province, China. BMC Infect Dis.

[90] Salman M, Steneroden K, Sing A (2022). Important zoonotic diseases of cattle and their prevention measures. Zoonoses: infections affecting humans and animals.

[91] Mburu CM, Bukachi SA, Tokpa KH, Fokou G, Shilabukha K, Ezekiel M (2021). Lay attitudes and misconceptions and their implications for the control of brucellosis in an agro-pastoral community in Kilombero district, Tanzania. PLoS Neglect Trop Dis.

[92] Mligo BJ, Sindato C, Yapi RB, Mathew C, Mkupasi EM, Kazwala RR (2022). Knowledge, attitude and practices of frontline health workers in relation to detection of Brucellosis in rural settings of Tanzania: a cross-sectional study. One Health Outlook.

[93] Al-homayani FK, Altalhi FM, Almalki ZA, Alnemari MA, Alfaifi HH, Alsaadi GK (2023). Public knowledge, attitudes, and practices regarding Brucellosis in Taif city, Saudi Arabia. Cureus.

[94] Sultana N, Pervin M, Sultana S, Mostaree M, Belal SMSH, Khan MAHNA (2022). Pathological investigation and molecular detection of bacterial zoonotic diseases of slaughtered cattle in Bangladesh. J Adv Biotechnol Exp Therap.

[95] Munsi MN, Akther S, Rahman MH, Hassan MZ, Ali MZ, Ershaduzzaman M (2021). Seroprevalence of Brucellosis in goats in some selected areas of Bangladesh. J Adv Vet Anim Res.

[96] Ahasan MS, Rahman MS, Rahman AKMA, Berkvens D (2017). Bovine and caprine Brucellosis in Bangladesh: bayesian evaluation of four serological tests, true prevalence, and associated risk factors in household animals. Trop Anim Health Prod.

[97] Khatun MM, Islam MA, Rahman MM (2019). Current status of veterinary public health activities in Bangladesh and its future plans. BMC Vet Res.

[98] Sayeed A, Faruque MR, Ahad A, Forhad Hossain M, Hoque MA (2020). Molecular detection and epidemiology of *Brucella* in dairy cattle of Bangladesh. Bangladesh J Vet Anim Sci J.

[99] Rahman AKMA, Saegerman C, Berkvens D (2016). Latent class evaluation of three serological tests for the diagnosis of human brucellosis in Bangladesh. Trop Med Health.

[100] Gani MO, Munsi MN, Ershaduzzaman M, Rahman AA, Sultana S, Alam MS (2016). Seroprevalence of ovine brucellosis in Bangladesh. Asian J Med Biol Res.

[101] Rahman AA (2015). Epidemiology of brucellosis in humans and domestic ruminants in Bangladesh.

[102] Hassan AA, Uddin MB, Islam MR, Cho HS, Hossain MM (2014). Serological prevalence of brucellosis of cattle in selected dairy farms in Bangladesh. Korean J Vet Res.

[103] Rahman AKMA, Saegerman C, Berkvens D, Fretin D, Gani MO, Ershaduzzaman M (2013). Bayesian estimation of true prevalence, sensitivity and specificity of indirect ELISA, rose Bengal test and slow agglutination test for the diagnosis of brucellosis in sheep and goats in Bangladesh. Prev Vet Med.

[104] Islam MA, Khatun MM, Werre SR, Sriranganathan N, Boyle SM (2013). A review of *Brucella* seroprevalence among humans and animals in Bangladesh with special emphasis on epidemiology, risk factors and control opportunities. Vet Microbiol.

[105] Sikder S, Mushfiqur SM, Md R, Alim A, Das S (2012). Haematological variations in *Brucella abortus* antibody positive cross-bred cattle at Chittagong, Bangladesh. YYU Vet Fakult Derg.

[106] Rahman AKMA, Dirk B, Fretin D, Saegerman C, Ahmed MU, Muhammad N (2012). Seroprevalence and risk factors for brucellosis in a high-risk group of individuals in Bangladesh. Foodborne Path Dis.

[107] Sikder S, Rahman AA, Faruque MR, Alim MA, Das S, Gupta A Das (2012). Bovine Brucellosis: an epidemiological study at Chittagong, Bangladesh. Pakistan Vet J.

[108] Uddin MJ, Rahman MS, Akter SH (2007). Brucellosis of goats (*Capra hircus*) in Bangladesh. J Bangladesh Agric Univ.

[109] Rahman MS, Han JC, Park J, Lee JH, Eo SK, Chae JS (2006). Prevalence of brucellosis and its association with reproductive problems in cows in Bangladesh. Vet Rec.

